# Metal Ion Chelates as Surrogates of Nucleobases for the Recognition of Nucleic Acid Sequences: The Pd^2+^ Complex of 2,6-Bis(3,5-dimethylpyrazol-1-yl)purine Riboside

**DOI:** 10.1155/2012/196845

**Published:** 2012-05-13

**Authors:** Sharmin Taherpour, Tuomas Lönnberg

**Affiliations:** Department of Chemistry, University of Turku, Vatselankatu 2, 20014 Turku, Finland

## Abstract

A 2,6-bis(3,5-dimethylpyrazol-1-yl)purine ribonucleoside has been prepared and incorporated as a conventionally protected phosphoramidite into a 9-mer 2′-*O*-methyl oligoribonucleotide. According to 1H NMR spectroscopic studies, this nucleoside forms with Pd^2+^ and uridine a ternary complex that is stable at a micromolar concentration range. CD spectroscopic studies on oligonucleotide hybridization, in turn, suggest that the Pd^2+^ chelate of this artificial nucleoside, when incorporated in a 2′-*O*-methyl-RNA oligomer, is able to recognize thymine within an otherwise complementary DNA strand. The duplex containing thymidine opposite to the artificial nucleoside turned out to be somewhat more resistant to heating than its counterpart containing 2′-deoxycytidine in place of thymidine, but only in the presence of Pd^2+^. According to UV-melting measurements, replacement of 2′-*O*-methyladenosine with the artificial nucleoside markedly enhances hybridization with a DNA target, irrespective of the identity of the opposite base and the presence of Pd^2+^. With the thymidine containing DNA target, the *T*_*m*_ value is 2–4°C higher than with targets containing any other nucleoside opposite to the artificial nucleoside, but the dependence on Pd^2+^ is much less clear than in the case of the CD studies.

## 1. Introduction

In nature, recognition of nucleic acid sequences is based on Watson-Crick base pairing, while vertical stacking of base pairs accounts for most of the duplex stability. At room temperature, 10 base pairs are typically required for a stable duplex, meaning that targeting sequences shorter than this by the conventional strategy, that is, using complementary oligonucleotides, is problematic. Sequences within a double-stranded region are also difficult to target because thermodynamics favor formation of the longer duplex. The first problem has partly been addressed by using small molecules exhibiting high affinity towards certain specific short sequences [1]. No clear-cut relationship exists between the target sequence and the structure of the small molecule, however, making the design and preparation of such molecules a demanding task. PNA oligonucleotides offer a solution to the second problem, as PNA/DNA heteroduplexes are more stable than native double-stranded DNA, but even in the best cases a substantial excess of the PNA is still needed for efficient invasion [2, 3].

Oligonucleotides composed of unnatural monomers with an enhanced affinity towards their complement nucleobases could potentially form stable duplexes even with short target sequences as well as invade double-stranded DNA even when present in only stoichiometric amounts. One way to achieve the desired high affinity is to exploit the coordination of a ring nitrogen (N1 of purines or N3 of pyrimidines) of the natural nucleobase to a soft metal ion, such as Pd^2+^, carried by an artificial nucleobase [4]. Discrimination between the four natural nucleobases, in turn, could be achieved through a combination of additional destabilizing (steric) and stabilizing (hydrogen bonding) interactions. A number of studies on metal-ion-mediated base pairs have been published [5, 6], but the focus has generally been on expanding the genetic code by introducing a completely new artificial base pair [7, 8] or impregnating DNA with metal ions for nanotechnological applications [9–11], rather than developing high-affinity complements for the natural nucleobases. In the former, both of the ligands of the metal-ion-mediated base pair may be freely designed for maximum performance (in many cases, the same ligand is used in both strands), whereas in the latter the sole artificial nucleobase must be able to not only coordinate the metal ion but also accommodate the steric and hydrogen bonding requirements of the natural nucleobase, presenting a unique challenge.

In the present study, the Pd^2+^complex of 2,6-bis-(3,5-dimethylpyrazol-1-yl)purine ribonucleoside **(1)** is used as an artificial nucleoside. Nucleoside **1**, with no potential for stabilizing hydrogen bond interactions, may be considered as a reference structure for future studies. On a monomeric level, the binding affinity and selectivity of this artificial nucleoside were studied by NMR titrations. For a more realistic model, nucleoside **1** was additionally converted to a phosphoramidite building block **(2)** and incorporated into a 2′-*O*-methyl-RNA oligonucleotide. The effect of Pd^2+^ on the secondary structure and thermal stability of duplexes between this modified oligonucleotide and complementary 2′-*O*-methyl-RNA and DNA oligonucleotides was investigated by melting temperature and CD spectrometric studies. For comparison, the same experiments were also performed on a 2′-*O*-methyl-RNA oligonucleotide having adenosine in the place of the artificial nucleoside.

## 2. Results

### 2.1. Synthesis of 2,6-bis(3,5-dimethylpyrazol-1-yl)purine Ribonucleoside **(1)** and Its Conversion to a Phosphoramidite Building Block **(2)**

**Scheme 1:**
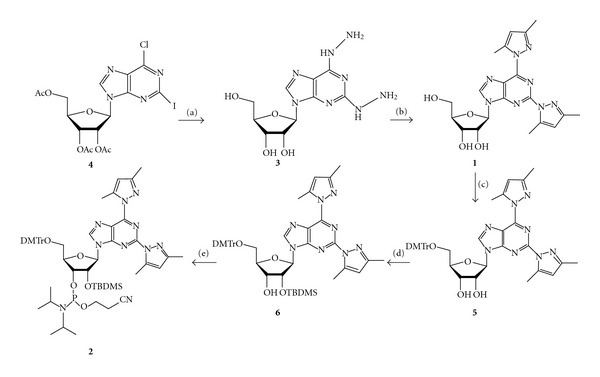
Preparation of the phosphoramidite building block **2**. Reagents and conditions: (a) H_2_NNH_2_·H_2_O; (b) acetylacetone, THF, TFA; (c) DMTrCl, pyridine; (d) TBDMSCl, imidazole, DMF; (e) 2-cyanoethyl-*N*, *N*-diisopropylchlorophosphoramidite, triethylamine, CH_2_Cl_2_.

Synthesis strategy for the phosphoramidite building block **2** is presented in Scheme 1. First, the 2,6-dihydrazinopurine riboside **3** was prepared by treating 2′,3′,5′-tri-*O-*acetyl-6-chloro-2-iodopurine riboside **(4)** with hydrazine hydrate at room temperature [12]. After that, the hydrazino groups were converted to 3,5-dimethylpyrazol-1-yl groups with 2,4-pentanedione [13] and the 5′-OH was protected as a 4,4′-dimethoxytrityl ether. Finally, the 2′-OH was silylated with TBDMSCl and the 3′-OH phosphitylated by the conventional methods.

### 2.2. Synthesis of Oligonucleotides

2′-*O*-Methyl-RNA oligonucleotides **7A, 8U**, **8A**, **8G**, **8C**,** 9U**, **9A**, **9G,** and **9C**, as well as the DNA oligonucleotides **10T**, **10A**, **10C,** and **10G**, were synthesized from commercial phosphoramidite building blocks by conventional phosphoramidite strategy on an automated synthesizer. The modified phosphoramidite monomer (**2**) was incorporated in the middle of a 9-mer 2′-*O*-Me-RNA sequence (**7X**) manually using elongated coupling time (60 min). The coupling yield for building block **2** was 36% and the subsequent couplings proceeded with normal (approximately 99%) efficiency. The crude oligonucleotides were purified by reversed-phase high-performance liquid chromatography (RP-HPLC) and characterized by electrospray ionization mass spectrometry (ESI-MS). The concentrations of the purified oligonucleotides were determined UV-spectrophotometrically using molar absorption coefficients calculated by an implementation of the nearest-neighbors method. The sequences of the oligonucleotides synthesized, as well as their RP-HPLC retention times, wavelengths of UV absorption maxima and observed and calculated molecular weights are summarized in Table 1.

### 2.3. NMR Spectrometric Titrations

The formation and stability of the complexes between the artificial nucleoside **1** and uridine in the presence and absence of Pd^2+^ were studied by ^1^H NMR spectrometric titration. The NMR spectra were recorded in a 120 mM deuterated phosphate buffer at pH 7.3. The initial concentration of the nucleoside components of the putative ternary complex, *namely,* **1** and uridine, was 3.6 mmol L^−1^, while a deficit of Pd^2+^ (0.7 eq., 2.5 mmol L^−1^ as K_2_PdCl_4_) was employed to ensure exclusive formation of the desired ternary complex. The titration was carried out by diluting the sample with buffer until the uridine concentration was 0.061 mmol L^−1^.

**Figure 1:**
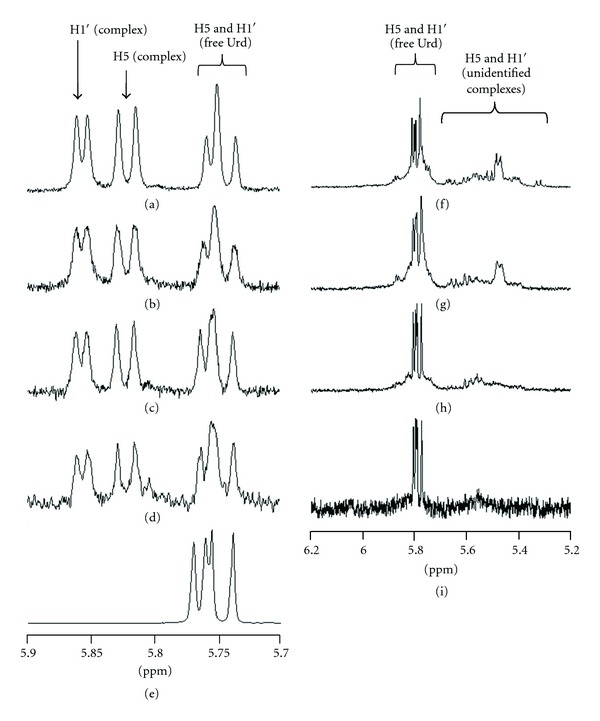
^1^H NMR spectra of uridine (H5 and H1′) at various concentrations of uridine, **1** and Pd^2+^ at pH 7.3. (a) [Urd] = [**1**] = 3.6 mmol L^−1^, [Pd^2+^] = 2.52 mmol L^−1^; (b) [Urd] = [**1**] = 1.49 mmol L^−1^, [Pd^2+^] = 1.04 mmol L^−1^;** (**c) [Urd] = [**1**] = 0.57 mmol L^−1^, [Pd^2+^] = 0.40 mmol L^−1^; (d): [Urd] = [**1**] = 0.061 mmol L^−1^, [Pd^2+^] = 0.042 mmol L^−1^; (e) [Urd] = [**1**] = 13.84 mmol L^−1^, [Pd^2+^] = 0; (f) [Urd] = 3.6 mmol L^−1^, [Pd^2+^] = 2.52 mmol L^−1^; (g) [Urd] = 1.49 mmol L^−1^, [Pd^2+^] = 1.04 mmol L^−1^;** (**h) [Urd] = 0.57 mmol L^−1^, [Pd^2+^] = 0.40 mmol L^−1^; (i) [Urd] = 0.061 mmol L^−1^.

**Figure 2:**
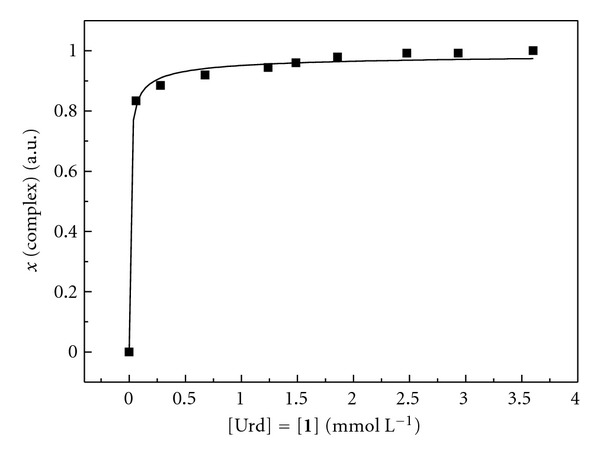
Mole fraction of uridine in complex as a function of the concentration of uridine and **1**; pH = 7.3.

Over the entire concentration range studied, two distinct sets of signals were observed for uridine: one referring to the free nucleoside and the other one (presumably) to the ternary complex (Figures 1(a)–1(d)). The two sets of signals were particularly well resolved in the case of H5 and H1′ resonance of uridine and the integrals of these signals were used to determine the mole fraction of the complex at each concentration (Figure 2). To exclude the possibility that the observed changes in the chemical shifts of the H5 and H1′ of uridine were caused by Pd^2+^ alone, a corresponding experiment with uridine and Pd^2+^ but without the artificial nucleoside **1** was carried out (Figures 1(f)–1(i)). In this case, an upfield change in the chemical shifts of the H5 and H1′ of uridine was observed, in striking contrast with the downfield shift associated with formation of the putative ternary complex. In other words, the H5 and H1′ signals assigned to uridine in its ternary complex with Pd^2+^ and the artificial nucleoside **1** were not observed in the absence of the latter. No effort was made to characterize the complexes between uridine and Pd^2+^ but the NMR spectra suggest that a number of different relatively weak complexes prevail, again in sharp contrast with the single strong complex formed in the presence of the artificial nucleoside **1**.

Upon introduction of Pd^2+^, the signals for the hydrogens on the pyrazole rings of **1** shift downfield by approximately 0.4 ppm. The signals of two of the methyl substituents of the pyrazole rings exhibit an even greater downfield shift (approximately 0.6 and 0.8 ppm, resp.), whereas the other two are shifted slightly upfield. Adding uridine to the system hardly affects the chemical shifts of these protons (data not shown).

**Figure 3:**
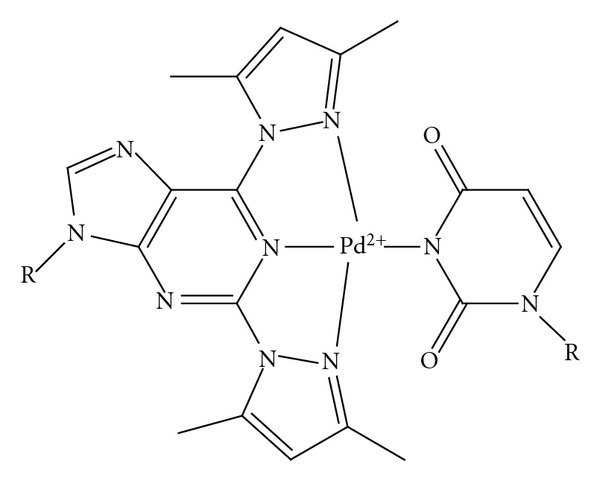
Proposed ternary complex between **1**, Pd^2+^, and uridine.

As expected, dilution of the sample was accompanied by decrease of the proportion of signals arising from the ternary complex (Figures 1(a)–1(d)). However, even at the lowest concentration employed, that is, 61 *μ*mol L^−1^, the mole fraction of the complex was still more than 80% of the saturation level observed at high concentrations. In the absence of Pd^2+^, no complex was formed even though a high concentration (13.84 mmol L^−1^) of the nucleosides was used (Figure 1(e)). In all likelihood, Pd^2+^ forms a tridentate chelate with the artificial nucleoside **1 **by binding to N1 of the purine ring and N2 of the pyrazolyl moieties, the fourth coordination site being filled by the N3 of uridine (Figure 3) [14]. The ability of thymine to form base pairs mediated by N3-coordinated soft metal ions has previously been demonstrated and uracil most likely exhibits similar behavior [15–19]. While no spectra could be obtained at sufficiently low concentrations to allow for reliable determination of the stability of the complex, the data at hand suggest a dissociation constant in the low micromolar range.

### 2.4. CD Spectropolarimetric Measurements

**Figure 4:**
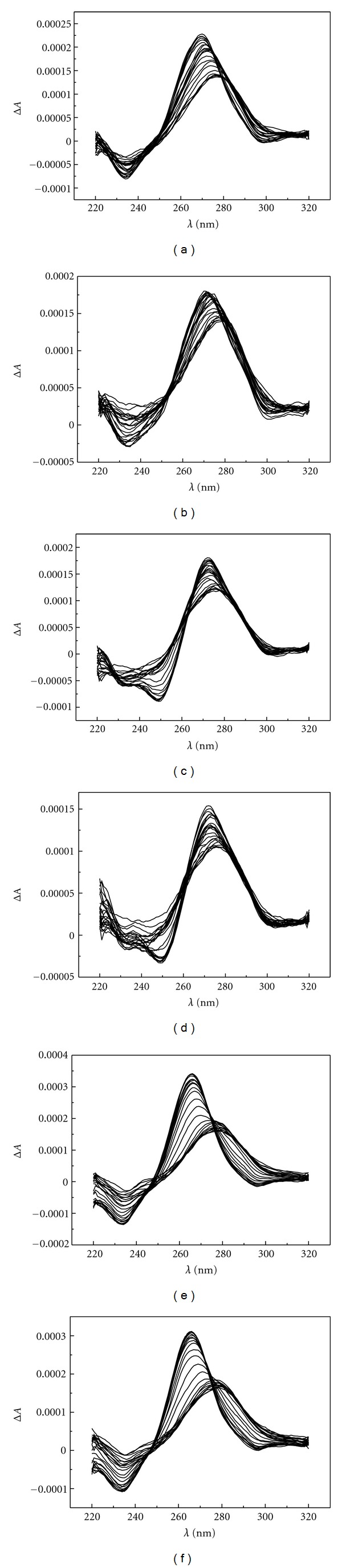
CD spectra of the duplexes formed between the modified (**7X**) or unmodified (**7A**) oligonucleotide and complementary DNA oligonucleotides having thymidine (**10T**) or 2′-deoxycytidine (**10C**) opposite to the artificial monomer (or adenosine) in the presence and absence of Pd^2+^; [oligonucleotides] = 3.0 *μ*M, [K_2_PdCl_4_] = 0/3.0 *μ*M, *I*(NaClO_4_) = 0.1 mol L^−1^, pH = 7.4. (a) **7X**:**10T**, (b) **7X**:**10T** + Pd^2+^, (c) **7X**:**10C**, (d) **7X**:**10C** + Pd^2+^, (e) **7A**:**10T**, (f) **7A**:**10T** + Pd^2+^.

To obtain information about the impact of the modified nucleoside and Pd^2+^ on the secondary structure of oligonucleotides, CD spectra of the duplexes of the modified (**7X**) and unmodified (**7A**) 2′-*O*-methyl oligoribonucleotides with complementary DNA or 2′-*O*-methyl-RNA oligonucleotides were measured over a wide temperature range (6–94°C) in the presence and absence of Pd^2+^. The experiments were carried out at pH 7.4 (20 mmol L^−1^ cacodylate buffer), the ionic strength being adjusted to 0.1 mol L^−1^ with NaClO_4_. The concentration of the oligonucleotides was 3.0 *μ*mol L^−1^ in each experiment and the concentration of Pd^2+^ (added as K_2_PdCl_4_) either 0 or 3.0 *μ*mol L^−1^. In all cases, at low temperatures the CD spectra are characteristic of an A-type duplex and almost all of these duplexes denature on increasing temperature, as evidenced by the gradual decrease of the CD signals [20–23]. The sole exception to this behavior is the duplex **7X**:**10T**, having thymidine opposite to the artificial nucleoside. This duplex seemed to be somewhat resistant to heating in the presence, but not in the absence, of Pd^2+^. Illustrative examples of the CD spectra obtained are presented in Figure 4 (see Supplementary Material available online at doi:10.1155/2012/196845). 

**Figure 5:**
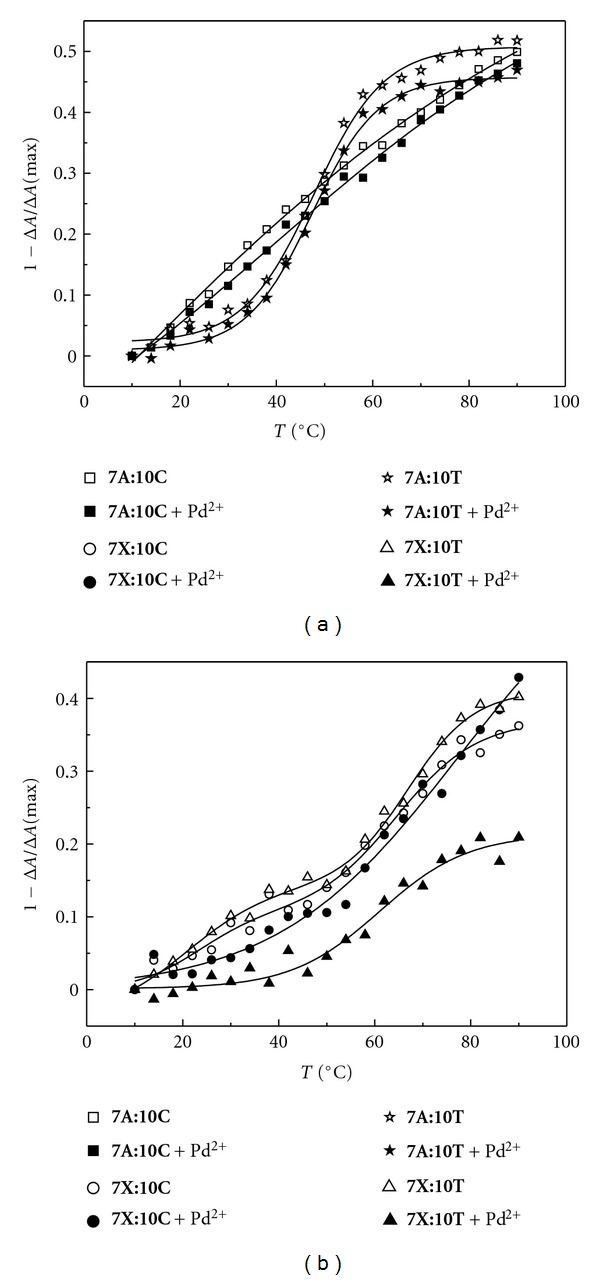
Temperature versus 1−Δ*A*/Δ*A*(max) profiles of the duplexes formed between the modified (**7X**) and unmodified (**7A**) oligonucleotides with the DNA oligonucleotides **10C **and** 10T** in the presence and absence of Pd^2+^; [oligonucleotides] = 3.0 *μ*M, [K_2_PdCl_4_] = 0/3.0 *μ*M, scan range 220–320 nm; *I*(NaClO_4_) = 0.1 mol L^−1^; pH = 7.4.

**Figure 6:**
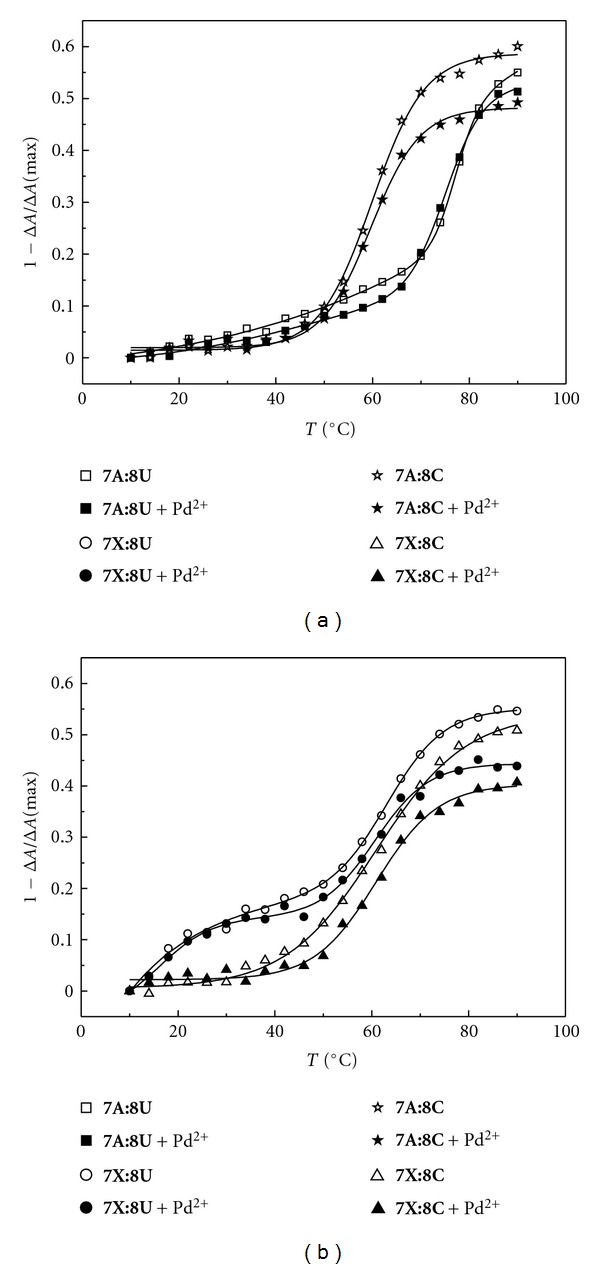
Temperature versus 1−Δ*A*/Δ*A*(max) profiles of the duplexes of the modified (**7X**) and unmodified (**7A**) oligonucleotides with the 2′-*O*-methyl-RNA oligoribonucleotides **8U** and **8C** in the presence and absence of Pd^2+^; [oligonucleotides] = 3.0 *μ*M, [K_2_PdCl_4_] = 0/3.0 *μ*M, scan range 220–320 nm; *I*(NaClO_4_) = 0.1 mol L^−1^; pH = 7.4.

**Figure 7:**
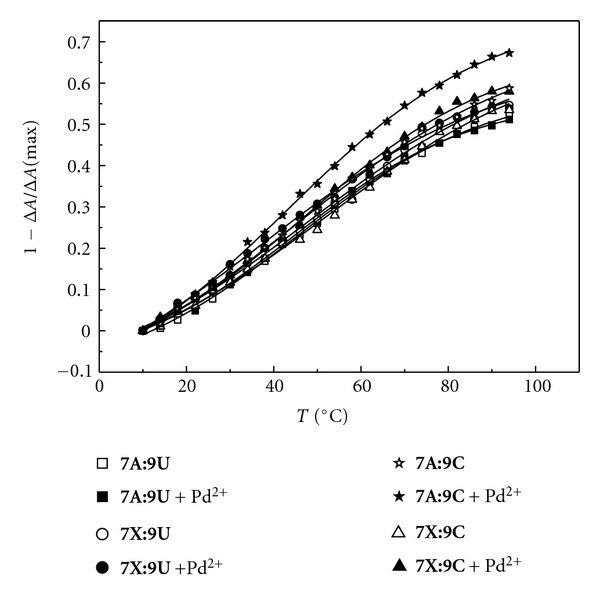
Temperature versus 1−Δ*A*/Δ*A*(max) profiles of the duplexes of the modified oligonucleotide **7X** or **7A **with oligoribonucleotide **9U **and **9C** in the presence and absence of Pd^2+^; [oligonucleotides] = 3.0 *μ*M, [K_2_PdCl_4_] = 0/3.0 *μ*M, scan range 220–320 nm; *I*(NaClO_4_) = 0.1 mol L^−1^; pH = 7.4.

The thermal loss of ellipticity for selected duplexes in the presence and absence of Pd^2+^ is presented in Figures 5–7. In the case of the 2′-*O*-methyl-RNA/DNA heteroduplexes, the unmodified full-match duplex **7A**:**10T** exhibits a sigmoid curve with an inflection point at approximately 50°C regardless of the presence of Pd^2+^ (Figure 5(a)). The curves for a corresponding mismatched duplex, **7A**:**10C**, also essentially overlap but are much more linear with no clear inflection point. With the modified oligonucleotide **7X**, the situation is quite different: the curves for the “mismatched” duplex **7X**:**10C** in the presence and absence of Pd^2+^ overlap with each other and also with the curve for the “full-match” duplex **7X**:**10T** in the absence of Pd^2+^ (Figure 5(b)). In the presence of Pd^2+^, however, duplex **7X**:**10T** is remarkably resistant to heating, losing significantly less of its ellipticity than the other combinations. In other words, the artificial 2,6-bis(3,5-dimethyl-1*H*-pyrazol-1-yl)purine nucleobase seems to be able to recognize thymine, but only in the presence of Pd^2+^.

With the 2′-*O*-methyl-RNA homoduplexes between unmodified oligonucleotides, the ellipticity of the matched duplex **7A**:**8U** exhibits a strongly sigmoid curve with an inflection point at approximately 75°C and the mismatched duplex **7A**:**8C** shows a similar curve with an inflection point at approximately 60°C (Figure 6(a)). The inflection points are essentially independent on the presence of Pd^2+^, but in the case of the mismatched duplex **7A**:**8C**, the loss of ellipticity at high temperatures is somewhat lower in the presence of Pd^2+^. In contrast, no discrimination between uridine and cytidine is observed with the modified oligonucleotide **7X**: both duplexes **7X**:**8U** and **7X**:**8C** exhibit largely similar sigmoid curves with inflection points at approximately 60°C (Figure 6(b)). In both cases, the maximum loss of ellipticity is somewhat lower in the presence of Pd^2+^. The plots of the duplexes containing multiple mismatches are essentially linear and independent on both the sequence and the presence of Pd^2+^ (Figure 7).

### 2.5. Melting Temperature Measurements

The melting temperatures of the duplexes formed between the modified oligonucleotide **7X** and complementary 2′-*O*-methyl-RNA or DNA sequences bearing A, G, U (or T), or C nucleoside opposite to the artificial nucleoside** 1 **were measured in the presence and absence of Pd^2+^. For comparison, the respective duplexes formed by the unmodified oligonucleotide **7A **were also studied. The *T*_*m*_ values were measured in a 20 mmol L^−1^ cacodylate buffer at pH 7.4, the ionic strength being adjusted to 0.1 mol L^−1^ with NaClO_4_. The concentration of the oligonucleotides was 3.0 *μ*mol L^−1^ in each experiment and the concentration of Pd^2+^ (added as K_2_PdCl_4_) either 0 or 3.0 *μ*mol L^−1^. The results of the *T*_*m*_ measurements are summarized in Table 2.

As expected, the unmodified oligonucleotide **7A** forms the most stable 2′-*O*-methyl-RNA/DNA heteroduplex with the fully complementary DNA oligonucleotide **10T**, but the discrimination is not very strict. For example, the full-match duplex **7A**:**10T** exhibits a *T*_*m*_ value of only 5°C higher than the mismatched duplex **7A**:**10A** (50.0 and 45.0°C, resp.). Adding 1 eq. of Pd^2+^ to the oligonucleotide mixtures has little effect on the stability of any of the duplexes studied. Unexpectedly, all of the respective heteroduplexes formed by the modified oligonucleotide **7X** turned out to be significantly more stable than the ones formed by **7A**, with *T*_*m*_ values ranging from 58.6 to 62.6°C. The most stable duplex was the one between **7X** and **10T**, although the selectivity was even lower than in the case of **7A**. Furthermore, the selectivity is only marginally improved in the presence of Pd^2+^.

In the case of the 2′-*O*-methyl-RNA homoduplexes, the unmodified oligonucleotide **7A** expectedly forms the most stable duplex with the fully oligonucleotide **8U** placing a uridine opposite to the central adenosine of **7A** (*T*_*m*_ = 75°C). Introducing a mismatch on both sides of this central base pair results in *T*_*m*_ a drop of 46°C with a matched central base pair (**7A**:**8U** → **7A **:** 9U**) and a drop of 12–17°C with a mismatched central base pair (**7A**:**8A** → **7A**:**9A**, **7A**:**8G** → **7A**:**9G**, and **7A**:**8C** → **7A**:**9C**). When the central adenosine of **7A** is replaced with the artificial nucleoside **1** (resulting in the modified oligonucleotide **7X**), *T*_*m*_ values of approximately 60°C are observed with all the homoduplexes studied, both in the presence and in the absence of Pd^2+^. In other words, the modification is strongly destabilizing compared to the full-match 2′-*O*-methyl-RNA homoduplex **7A**:**8U**, neither stabilizing nor destabilizing in the 2′-*O*-methyl-RNA homoduplexes containing a single mismatch and, unexpectedly, strongly stabilizing in the 2′-*O*-methyl-RNA homoduplexes containing multiple mismatches (as well as in the 2′-*O*-methyl-RNA/DNA heteroduplexes).

## 3. Discussion

The Pd^2+^ chelate of the 2,6-bis-(3,5-dimethylpyrazol-1-yl)purine nucleobase presented in this study has no potential for hydrogen bonding interactions with the canonical nucleobases—in other words, its base pairing relies solely on metal ion coordination. On the other hand, the modified nuclobase presents a much greater surface for *π*-*π* stacking interactions and the observed Pd^2+^-independent stabilization of the mismatched and 2′-*O*-methyl-RNA/DNA heteroduplexes is probably a result of this enhanced base-stacking. The Pd^2+^-dependent selective recognition of a thymine base within a complementary DNA oligonucleotide, in turn, is probably attributable to two factors, *namely,* the relatively small size of the thymine base, as well as its potential to act as an anionic nitrogen ligand. Of the four native nucleobases, only the pyrimidines may form a base pair of Watson-Crick geometry with the artificial metallo-nucleobase (and even these base pairs would be approximately 1.3 Å longer than the natural ones). Of the two pyrimidine nucleobases, only thymine (or uracil) is readily deprotonated to yield a negatively charged nitrogen ligand with a significantly greater affinity for metal ions. Indeed, Hg^II^ has been shown to selectively bind to a T-T mispair within a double-stranded oligonucleotide by coordinating to the N3 of both thymine residues [16–19]. The fact that no selectivity for uracil is observed with the 2′-*O*-methyl-RNA homoduplexes may be reasoned if the bulky dimethylpyrazole moieties cause a large enough steric hindrance to prevent the metallo-base pair from assuming planar geometry. Presumably, a nonplanar base pair should be more easily accommodated within the more flexible 2′-*O*-methyl-RNA/DNA heteroduplex than the relatively rigid 2′-*O*-methyl-RNA homoduplex. 

## 4. Conclusions

A 2′-*O*-methyl-RNA oligonucleotide incorporating an artificial nucleobase that serves as a tridentate chelate for soft metal ions has been synthesized. When hybridized with a complementary DNA oligonucleotide, this modified oligonucleotide selectively recognizes a thymine residue opposite to the artificial nucleobase. The discrimination is somewhat more pronounced in the presence of 1 eq. of Pd^2+^, but the difference is too small to be taken as compelling evidence for the formation of a metal-ion-mediated base pair between thymidine and **1**. Within fully matched 2′-*O*-methyl-RNA homoduplexes, the modification is destabilizing regardless of the presence of Pd^2+^, probably because the proposed metallo-base pair cannot assume a planar geometry needed to fit within the relatively tight base stack of these oligonucleotides. Within mismatched duplexes, as well as 2′-*O*-methyl-RNA/DNA heteroduplexes, the modification is stabilizing but the effect is rather sequence and Pd^2+^ independent, suggesting that the origin of this stabilization is the large *π*-*π* stacking surface of the artificial nucleobase. 

The metal-ion-carrying nucleobase presented in this study represents a reference structure with no potential of specific interactions with the natural nucleobases and the preference for thymine is probably based on its small size and ability to serve as an anionic nitrogen ligand. The fact that the strong base pairing observed at the monomeric level is only modestly reflected in the thermal stability of the respective double-stranded oligonucleotide is most likely due to difficulties in accommodating this (presumably) non-planar base pair within an oligonucleotide environment. It should be noted that the CD and *T*_*m*_ studies do not provide first-hand information on the interactions (if any) between thymine (or uracil) and the Pd^2+^ complex of the artificial nucleoside **1**. In other words, the possibility that within a double-stranded oligonucleotide, a complex of a different geometry prevails cannot be completely ruled out. More elaborate structures featuring additional hydrogen bonding interactions as well as carefully placed steric constraints will, in all likelihood, exhibit greatly enhanced affinity and selectivity. For example, replacing the dimethylpyrazole moieties with monosubstituted hydrazines would result in a structure not only sterically less hindered than **1** but also capable of donating two hydrogen bonds to the oxo substituents of thymine or uracil. On the other hand, using a pyrimidine, rather than a purine, nucleoside as the parent compound would alleviate some of the steric crowding caused by the relatively bulky Pd^2+^ ion. Finally, other soft metal ions could be screened to find the optimal combination of affinity and selectivity.

## 5. Experimental

### 5.1. General

2′,3′,5′-Tri-*O*-acetyl-6-chloro-2-iodopurine riboside was a commercial product that was used as received. For the triethylammonium acetate buffer used in HPLC chromatography, triethylamine was distilled before using. The NMR spectra were recorded with a Bruker Avance 500 NMR spectrometer and the chemical shifts are given in ppm. The mass spectra were recorded with a Bruker micrOTOF-Q ESI-MS spectrometer and the CD spectra with an Applied Photophysics Chirascan spectropolarimeter. The oligonucleotides were assembled on an Applied Biosystems 3400 DNA/RNA synthesizer. 

### 5.2. 9-(*β*-d-Ribofuranosyl)-2,6-dihydrazinopurine **(3)**

To neat hydrazine hydrate (6.5 mL, 134 mmol) was added 2′,3′,5′-tri-*O*-acetyl-6-chloro-2-iodopurine riboside (1.03 g, 1.91 mmol) and the resulting mixture was stirred for 14 d at room temperature. Over the course of the first day, the appearance of the reaction mixture changed from yellowish and cloudy to clear and colorless to light red, followed by formation of a white precipitate. The reaction mixture was diluted with 2-propanol (15 mL) and stirred for 15 min, after which the precipitate was collected by filtration and washed with cold 2-propanol (2 × 100 mL). Finally, the precipitate was dried under vacuum to yield 0.534 g (89%) of **2** as a white powder. ^1^H NMR (500 MHz, DMSO-*d*_6_) *δ*_*H*_ 7.92 (s, 1H, H8), 5.76 (d, 1H, H1′, *J *= 6.3 Hz), 4.56 (dd, 1H, H2′, *J*_1_ = 5.35, *J*_2_ = 5.50 Hz), 4.11 (dd, 1H, H3′, *J*_1_ = 3.1 Hz, *J*_2_ = 4.95), 3.89 (m, 1H, H4′), 3.61 (dd, 1H, H5′  *J*_1_ = 3.9 Hz, *J*_2_ =11.9 Hz), 3.52 (dd, 1H, H5′′  *J*_1_ = 3.9 Hz, *J*_2_ =11.9 Hz). ^13^C NMR (125 MHz, DMSO) *δ*_*C*_ 162.2, 155.9, 136.8, 136.7, 113.5, 87.4, 85.9, 73.6, 71.2, 62.2. HRMS (ESI^+^): *m/z *calcd 313.1367 obsd 313.1378 [M+H]^+^.

### 5.3. 9-(*β*-d-Ribofuranosyl)-2,6-bis(3,5-dimethylpyrazol-1-yl)purine **(1)**

To dry compound **3** (0.337 g, 1.08 mmol) was added 6.4 eq. of dry 2,4-pentanedione (0.70 mL, 6.912 mmol) and 0.1 eq. of trifluoroacetic acid (8.27 *μ*L, 0.108 mmol). The reaction mixture was stirred at room temperature for 16 h, during which time the product crystallized. The volatiles were removed under reduced pressure to yield crude **1** that was used in the next step without further purification. For the NMR titrations, a small sample was purified by silica gel chromatography eluting with a mixture of MeOH, CH_2_Cl_2_ and Et_3_N (9 : 90 : 1, *v*/*v*). ^1^H NMR (500 MHz, D_2_O) *δ*_*H*_ 8.46 (s, 1H, H8), 6.02 (d, 1H, H1′, *J *= 5.0 Hz), 6.0 (s, 1H, pyrazole), 5.88 (s, 1H, pyrazole), 4.61 (dd, 1H, H2′, *J*_1_ = *J*_2_ = 5.1 Hz), 4.27 (dd, 1H, H3′, *J*_1_ = 5.1 Hz, *J*_2_ = 5.0 Hz), 4.1 (m, 1H, H4′), 3.78 (dd, 1H, H5′, *J*_1_ = 2.7 Hz, *J*_2_ =12.8 Hz), 3.7 (dd, 1H, H5′′, *J*_1_ = 2.7 Hz, *J*_2_ = 12.8 Hz), 2.30 (s, 3H, CH_3_), 2.28 (s, 3H, CH_3_), 2.12 (s, 3H, CH_3_), 2.04 (s, 3H, CH_3_). ^13^C NMR (125 MHz, D_2_O) *δ*_*C*_ 153.9, 153.8, 152.1, 150.5, 149.6, 146.8, 143.8, 143.5, 107.5, 106.4, 70.5, 68.0, 67.8, 28.9, 27.9, 23.1, 13.8, 13.0, 12.5, 12.3. HRMS (ESI^+^): *m/z* calcd 441.1993 obsd 441.2036 [M + H]^+^.

### 5.4. 9-[5-O-(4,4′-Dimethoxytrityl)-*β*-d-ribofuranosyl]-2,6-bis(3,5-dimethylpyrazol-1-yl)purine **(5)**

To a solution of crude compound **1** (0.477 g, 1.08 mmol) in dry pyridine (10 mL) was added 1.6 eq. of DMTrCl (0.589 g, 1.738 mmol). The reaction mixture was stirred for 4 d at room temperature, after which it was concentrated under reduced pressure. The residue was dissolved in CH_2_Cl_2_ (40 mL) and washed with saturated aq. NaHCO_3_ (50 mL). The organic phase was dried with Na_2_SO_4_ and evaporated to dryness. The residue was purified by silica gel chromatography eluting with a mixture of EtOAc, hexane, and Et_3_N (stepwise gradient from 60 : 39 : 1 to 99 : 0 : 1, *v*/*v*). Yield 0.765 g (82.7%). ^1^H NMR (500 MHz, CDCl_3_) *δ*_*H*_ 8.36 (s, 1H, H8), 7.24 (m, 9H, Ar), 6.77 (m, 4H, Ar), 6.43 (d, 1H, H1′, *J *= 6.4 Hz), 6.11 (s, 1H, pyrazole), 6.05 (s, 1H, pyrazole), 4.74 (dd, 1H, H2′, *J*_1_ = 5.4 Hz, *J*_2_ = 5.5 Hz), 4.42 (m, 2H, H3′ & H4′), 3.76 (s, 6H, OCH_3_), 3.44 (dd, 1H, H5′, *J*_1_ = 3.4 Hz, *J*_2_ =10.5 Hz), 3.27 (dd, 1H, H5′′, *J*_1_= 3.4 Hz, *J*_2_ = 10.5 Hz), 2.66 (s, 3H, CH_3_), 2.63 (s, 3H, CH_3_), 2.39 (s, 3H, CH_3_), 2.27 (s, 3H, CH_3_). ^13^C NMR (125 MHz, CDCl_3_) *δ*_*C*_ 158.5, 153.1, 151.2, 150.7, 150.4, 149.8, 149.5, 144.4, 143.2, 142.8, 142.7, 135.5, 130.1, 128.1, 127.9, 126.9, 123.8, 122.9, 113.3, 110.5, 110.1, 88.9, 86.6, 85.6, 76.1, 72.2, 63.5, 55.2, 14.8, 14.1, 13.9, 13.8. HRMS (ESI^+^): *m/z* calcd 765.3227 obsd 765.3125 [M + Na]^+^. 

### 5.5. 9-[5-O-(4,4′-Dimethoxytrityl)-2-O-tert-butyldimethylsilyl-*β*-d-ribofuranosyl]-2,6-bis(3,5-dimethylpyrazol-1-yl)purine **(6)**

To a solution of dry compound **5** (0.543 g, 0.731 mmol) in dry DMF (10 mL) was added a solution of dry imidazole (0.5 g, 7.34 mmol) in DMF (15 mL), followed by 2.63 eq. of TBDMSCl (0.29 g, 1.92 mmol). After being stirred for 4 days, the reaction was quenched by adding MeOH (3 mL). Stirring was continued for 7 min, after which EtOAc (40 mL) and water (50 mL) were added and the organic and aqueous phases separated. The organic phase was dried with Na_2_SO_4_ and evaporated to dryness. The residue was purified by silica gel chromatography eluting with a mixture of EtOAc, CH_2_Cl_2_, and Et_3_N (stepwise gradient from 10 : 89 : 1 to 20 : 79 : 1,* v*/*v*). Yield 0.527 g (84%). ^1^H NMR (500 MHz, CDCl_3_) *δ*_*H*_ 8.37 (s, 1H, H8), 7.31 (m, 9H, Ar), 6.82 (m, 4H, Ar), 6.23 (d, 1H, H1′, *J *= 5.9 Hz), 6.10 (s, 1H, pyrazole), 6.04 (s, 1H, pyrazole), 4.79 (dd, 1H, H2′, *J*_1_ = 5.5 Hz, *J*_2_ = 5.6 Hz), 4.32 (m, 1H, H3′), 4.27 (m, 1H, H4′), 3.77 (s, 6H, OCH_3_), 3.52 (dd, 1H, H5′, *J*_1_ = 3.5 Hz, *J*_2_ = 9.2 Hz), 3.38 (dd, 1H, H5′′, *J*_1_ = 2.4 Hz, *J*_2_ = 9.2 Hz), 2.82 (s, 3H, CH_3_), 2.65 (s, 3H, CH_3_), 2.40 (s, 3H, CH_3_), 2.33 (s, 3H, CH_3_), 0.82 (s, 9H, Si–CCH_3_), 0.00 (s, 3H, SiCH_3_), −0.17 (s, 3H, SiCH_3_). ^13^C NMR (125 MHz, CDCl_3_) *δ*_*C*_ 158.6, 154.6, 153.4, 151.5, 150.8, 150.1, 144.4, 144.2, 142.5, 142.2, 135.4, 130.0, 128.0, 127.0, 121.8, 113.3, 110.7, 109.8, 87.3, 86.8, 84.2, 76.7, 71.7, 63.6, 55.2, 25.7, 25.5, 17.9, 15.0, 14.3, 13.9, −4.8, −5.1. HRMS (ESI^+^): *m/z* calcd 879.4092 obsd 879.4038 [M + Na]^+^. 

### 5.6. 9-{5-O-(4,4′-Dimethoxytrityl)-2-O-tert-butyldimethylsilyl-3-O-[(2-cyanoethoxy)-(N,N-di-isopropylamino)phosphinyl]-*β*-d-ribofuranosyl}-2,6-bis(3,5-dimethylpyrazol-1-yl)purine **(2)**

To a solution of dry compound **6** (114 mg, 0.133 mmol) in dry DCM (4 mL) was added 9 eq. of dry Et_3_N (0.167 mL, 1.197 mmol) and 2.5 eq. of 2-cyanoethyl-*N*,*N*-diisopropylchlorophosphoramidite (75 *μ*L, 0.333 mmol). The mixture was stirred under nitrogen atmosphere for 3 days, after which the reaction was quenched with MeOH (200 *μ*L). Stirring was continued for 10 min, after which CH_2_Cl_2_ (40 mL) was added and the resulting solution washed with saturated aq. NaHCO_3_ (40 mL). The organic phase was dried with Na_2_SO_4_ and evaporated to dryness. The residue was purified by silica gel chromatography eluting with a mixture of EtOAc, CH_2_Cl_2_, and Et_3_N (10 : 89 : 1,* v*/*v*). Yield 0.111 g (79%). ^1^H NMR (500 MHz, CDCl_3_) *δ*_*H*_ 8.41 (s, 1H, H8), 7.33 (m, 9H, Ar), 6.83 (m, 4H, Ar), 6.29 (m, 1H, H1′), 6.10 (s, 1H, pyrazole), 6.04 (s, 1H, pyrazole), 4.82 (dd, 1H, H2′, *J*_1_ = *J*_2_ = 5.50 Hz), 4.35 (m, 2H, H3′ & H4′), 3.78 (s, 6H, OCH_3_), 3.62 (m, 2H, CH_2_), 3.60 (m, 1H, H5′), 3.50 (m, 1H, H5′′), 2.86 (s, 3H, CH_3_), 2.67 (s, 3H, CH_3_), 2.64 (s, 2H, CH), 2.40 (s, 3H, CH_3_), 2.33 (s, 3H, CH_3_), 2.27 (m, 2H, CH_2_), 1.19 (d, 6H, CH_3_, *J* = 7.5 Hz), 1.05 (d, 6H, CH_3_, *J* = 8.3 Hz), 0.75 (s, 9H, Si–CCH_3_), 0.00 (s, 3H, SiCH_3_), −0.16 (s, 3H, SiCH_3_). ^13^C NMR (125 MHz, CDCl_3_) *δ*_*C*_ 158.6, 154.9, 153.2, 151.5, 150.8, 150.1, 144.3, 144.2, 142.5, 142.4, 142.1, 135.3, 130.1, 130.0, 128.1, 128.0, 127.1, 121.8, 117.5, 113.3, 110.6, 109.8, 86.9, 86.8, 84.7, 84.1, 72.9, 63.5, 58.9, 58.8, 55.2, 43.0, 25.6, 24.6, 20.5, 18.0, 15.0, 14.9, 14.3, 13.9, −4.6, −5.0, −5.1. ^31^P NMR (162 MHz, CDCl_3_) *δ*_*P*_ 152.1, 148.9. HRMS (ESI^+^):* m/z *calcd 1057.5218 obsd 1057.5188 [M + H]^+^. 

### 5.7. Oligonucleotide Synthesis

The oligonucleotides were assembled on a CPG-supported succinyl linker at a loading of 27 mmol g^−1^. Standard phosphoramidite strategies for RNA (600 s coupling time) or DNA (20 s coupling time) were used throughout the sequences, except for the modified phosphoramidite building block **2**, which was coupled manually using an increased coupling time (60 min). Based on trityl response, coupling yield for building block **2** was 36% and the other couplings proceeded with normal efficiency. The products were released from support and deprotected by conventional treatment with 33% aq. NH_3_ (5 hours at 55°C). The crude oligonucleotides were purified by RP-HPLC on a Hypersil ODS column C18 (250 × 4.6 mm, 5 *μ*m) eluting with a mixture of 0.10 mol L^−1^ aq. triethylammonium acetate and MeCN, the flow rate being 1.0 mL min^−1^. The amount of MeCN was increased linearly from 10 to 40% during 25 min for the 2′-*O*-methyl-RNA oligonucleotides and from 10 to 30% during 25 min for the DNA oligonucleotides. The purified products were characterized by ESI-MS analysis. 

## Supplementary Material

Supporting Information for: Metal ion chelates as surrogates of nucleobases for the recognition of nucleic acid sequences: the pD2*+* complex of 2,6-bis(3,5-dimethylpyrazol-1-yl)purine riboside.

## Figures and Tables

**Table 1:** The sequences, RP-HPLC retention times, wavelengths of UV absorption maxima, and observed and calculated molecular weights of the oligonucleotides prepared.

Sequence		*t*_ R_ /minute^a^	*λ*/nm	*m/z* obsd.	*m/z* calcd.
**7X**	5′-GCGC**X**CCGG-3^′b^	16.0	267.8	3152.8	3152.7
**7A**	5′-GCGC**A**CCGG-3′	16.1	257.6	2993.6	2993.8
**8U**	3′-CGCG**U**GGCC-5′	17.2	258.4	2970.6	2970.8
**8A**	3′-CGCG**A**GGCC-5′	17.5	257.6	2993.6	2993.8
**8G**	3′-CGCG**G**GGCC-5′	14.1	257.2	3009.6	3009.8
**8C**	3′-CGCG**C**GGCC-5′	16.6	258.8	2969.6	2969.8
**9U**	3′-CGC**AUA**GCC-5′	11.8	260.2	2938.6	2938.8
**9A**	3′-CGC**AAA**GCC-5′	12.3	259.1	2961.6	2961.8
**9G**	3′-CGC**AGA**GCC-5′	11.9	261.0	2977.6	2977.8
**9C**	3′-CGC**ACA**GCC-5′	12.4	267.0	2937.6	2937.8
**10T**	3′-d(CGCG**T**GGCC)-5′	10.8	258.0	2714.3	2714.8
**10A**	3′-d(CGCG**A**GGCC)-5′	10.0	257.2	2723.4	2723.8
**10C**	3′-d(CGCG**C**GGCC)-5′	11.0	271.2	2699.3	2699.8
**10G**	3′-d(CGCG**G**GGCC)-5′	10.5	256.0	2739.4	2739.8

^a^See Section 5 for the HPLC conditions. ^b^X refers to the artificial nucleoside **1**.

**Table 2:** The *T*_*m*_ of the duplexes formed between modified **(7X)** or unmodified **(7A)** oligonucleotide and complementary 2′-*O*-methyl-RNA (or DNA) sequences bearing A, G, U (or T), or C nucleoside opposite to the artificial nucleoside **1** (in the case of oligonucleotide **7X**) or adenosine (in the case of oligonucleotide **7A**) in the presence and absence of Pd^2+^; [oligonucleotides] = 3.0 *μ*M, [K_2_PdCl_4_] = 0/3.0 *μ*M, *I*(NaClO_4_) = 0.1 M; pH = 7.4.

Complementary oligonucleotide	Sequence	*T*_*m*_/°C, duplex with **7X**		*T*_*m*_/°C, duplex with **7A**	
		(5′-GCGCXCCGG-3′)		(5′-GCGCACCGG-3′)	
		**Pd^2+^**		**Pd^2+^**	
		**+**	**−**	**+**	**−**
**8U**	3′-CGCG**U**GGCC-5′	60.6 ± 0.6	61.8 ± 0.4	73.7 ± 0.1	75.1 ± 0.3
**8A**	3′-CGCG**A**GGCC-5′	61.6 ± 0.1	61.4 ± 0.4	56.3 ± 0.1	56.3 ± 0.2
**8G**	3′-CGCG**G**GGCC-5′	60.1 ± 0.6	61.6 ± 0.3	60.4 ± 0.1	61.2 ± 0.3
**8C**	3′-CGCG**C**GGCC-5′	59.9 ± 0.4	60.7 ± 0.2	58.8 ± 0.2	59.0 ± 0.2
**9U**	3′-CGC**AUA**GCC-5′	60.4 ± 0.1	60.4 ± 0.3	27.6 ± 0.1	26.9 ± 0.3
**9A**	3′-CGC**AAA**GCC-5′	61.7 ± 0.9	61.0 ± 0.9	44.3 ± 0.6	43.6 ± 0.7
**9G**	3′-CGC**AGA**GCC-5′	60.1 ± 0.2	61.8 ± 0.4	43.5 ± 0.9	44.3 ± 0.3
**9C**	3′-CGC**ACA**GCC-5′	61.1 ± 0.3	60.3 ± 0.5	43.9 ± 0.7	45.7 ± 0.8
**10T**	3′-d(CGCG**T**GGCC)-5′	62.6 ± 1.3	60.8 ± 0.2	49.5 ± 0.5	50.0 ± 0.1
**10A**	3′-d(CGCG**A**GGCC)-5′	59.1 ± 0.9	60.5 ± 0.5	43.5 ± 0.4	45.0 ± 0.3
**10C**	3′-d(CGCG**C**GGCC)-5′	60.7 ± 0.2	61.6 ± 0.1	45.5 ± 0.1	50.3 ± 0.2
**10G**	3′-d(CGCG**G**GGCC)-5′	58.6 ± 0.4	60.2 ± 0.2	40.8 ± 0.9	43.9 ± 0.5
