# Supplementary material for: Metal Ion Chelates as Surrogates of Nucleobases for the Recognition of Nucleic Acid Sequences: The Pd2+ Complex of 2,6-Bis(3,5-dimethylpyrazol-1-yl)purine Riboside
Source: J Nucleic Acids. 2012 May 13;2012:196845. doi: 10.1155/2012/196845 (PMC3359700; doi:10.1155/2012/196845)
Supplement: Supplementary file 1 — Supporting Information for: Metal ion chelates as surrogates of nucleobases for the recognition of nucleic acid sequences: the pD2+ complex of 2,6-bis(3,5-dimethylpyrazol-1-yl)purine riboside. [file 196845.f1.pdf]

Supporting Information for:

METAL ION CHELATES AS SURROGATES OF NUCLEOBASES FOR THE RECOGNITION OF NUCLEIC ACID SEQUENCES: THE Pd^{2+} COMPLEX OF 2,6-BIS(3,5-DIMETHYLPYRAZOL-1-YL)PURINE RIBOSIDE

Sharmin Taherpour and Tuomas Lönnberg*

Department of Chemistry, University of Turku, Vatselankatu 2, FIN-20014, Turku, Finland

Email: tuanlo@utu.fi

Contents:

T vs. 1- ΔA/ΔA(max) profiles of the heteroduplexes formed between **7A** and DNA oligonucleotides…………………………………… S3

T vs. 1- ΔA/ΔA(max) profiles of the heteroduplexes formed between **7X** and DNA oligonucleotides…………………………………… S4

T vs. 1- ΔA/ΔA(max) profiles of the homoduplexes formed between **7A** and 2´-*O*-methyl-RNA oligonucleotides……………………… S5

T vs. 1- ΔA/ΔA(max) profiles of the homoduplexes formed between **7A** and 2´-*O*-methyl-RNA oligonucleotides……………………… S6

T vs. 1- ΔA/ΔA(max) profiles of the mismatched homoduplexes formed between **7A** and 2´-*O*-methyl-RNA oligonucleotides……….. S7

T vs. 1- ΔA/ΔA(max) profiles of the mismatched homoduplexes formed between **7A** and 2´-*O*-methyl-RNA oligonucleotides……….. S8

CD spectra of the homoduplex **7X**:**8U** in the absence and presence of Pd^{2+}……………………………………………………………. S9

CD spectra of the homoduplex **7A**:**8U** in the absence and presence of Pd^{2+}……………………………………………………………. S10

CD spectra of the homoduplex **7X**:**8C** in the absence and presence of Pd^{2+}……………………………………………………………. S11

CD spectra of the homoduplex **7A**:**8C** in the absence and presence of Pd^{2+}……………………………………………………………. S12

CD spectra of the homoduplex **7X**:**8G** in the absence and presence of Pd^{2+}……………………………………………………………. S13

CD spectra of the homoduplex **7A**:**8G** in the absence and presence of Pd^{2+} …… S14

CD spectra of the homoduplex **7X**:**8A** in the absence and presence of Pd^{2+} …… S15

CD spectra of the homoduplex **7A**:**8A** in the absence and presence of Pd^{2+} …… S16

CD spectra of the heteroduplex **7X**:**10A** in the absence and presence of Pd^{2+} …… S17

CD spectra of the heteroduplex **7A**:**10A** in the absence and presence of Pd^{2+} …… S18

CD spectra of the heteroduplex **7X**:**10C** in the absence and presence of Pd^{2+} …… S19

CD spectra of the heteroduplex **7A**:**10C** in the absence and presence of Pd^{2+} …… S20

CD spectra of the heteroduplex **7X**:**10G** in the absence and presence of Pd^{2+} …… S21

CD spectra of the heteroduplex **7A**:**10G** in the absence and presence of Pd^{2+} …… S22

CD spectra of the mismatched homoduplex **7X**:**9A** in the absence and presence of Pd^{2+} …… S23

CD spectra of the mismatched homoduplex **7A**:**9A** in the absence and presence of Pd^{2+} …… S24

CD spectra of the mismatched homoduplex **7X**:**9C** in the absence and presence of Pd^{2+} …… S25

CD spectra of the mismatched homoduplex **7A**:**9C** in the absence and presence of Pd^{2+} …… S26

CD spectra of the mismatched homoduplex **7X**:**9G** in the absence and presence of Pd^{2+} …… S27

CD spectra of the mismatched homoduplex **7A**:**9G** in the absence and presence of Pd^{2+} …… S28

CD spectra of the mismatched homoduplex **7X**:**9U** in the absence and presence of Pd^{2+} …… S29

CD spectra of the mismatched homoduplex **7A**:**9U** in the absence and presence of Pd^{2+} …… S30

ESI^- mass spectrum of the modified oligonucleotide **7X** …… S31

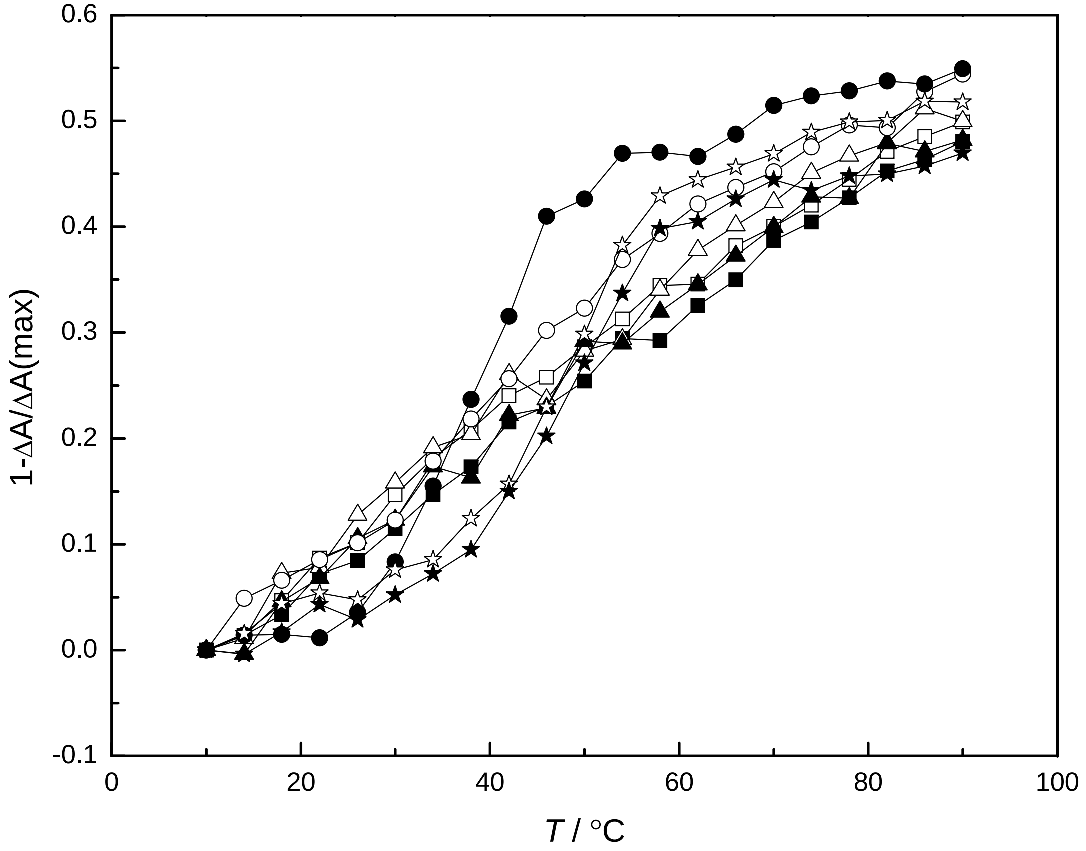

Figure S1. Temperature vs. 1- ΔA/ΔA(max) profiles of the heteroduplexes formed between **7A** and DNA oligonucleotides in the presence and absence of Pd^{2+}; scan range 220 – 320 nm; $I(NaClO_4)$ = 0.1 M; pH = 7.4. (□) **7A**:**10C**, (■) **7A**:**10C** + Pd^{2+}, (○) **7A**:**10G**, (●) **7A**:**10G** + Pd^{2+}, (☆) **7A**:**10T**, (★) **7A**:**10T** + Pd^{2+}, (△) **7A**:**10A**, (▲) **7A**:**10A** + Pd^{2+}.

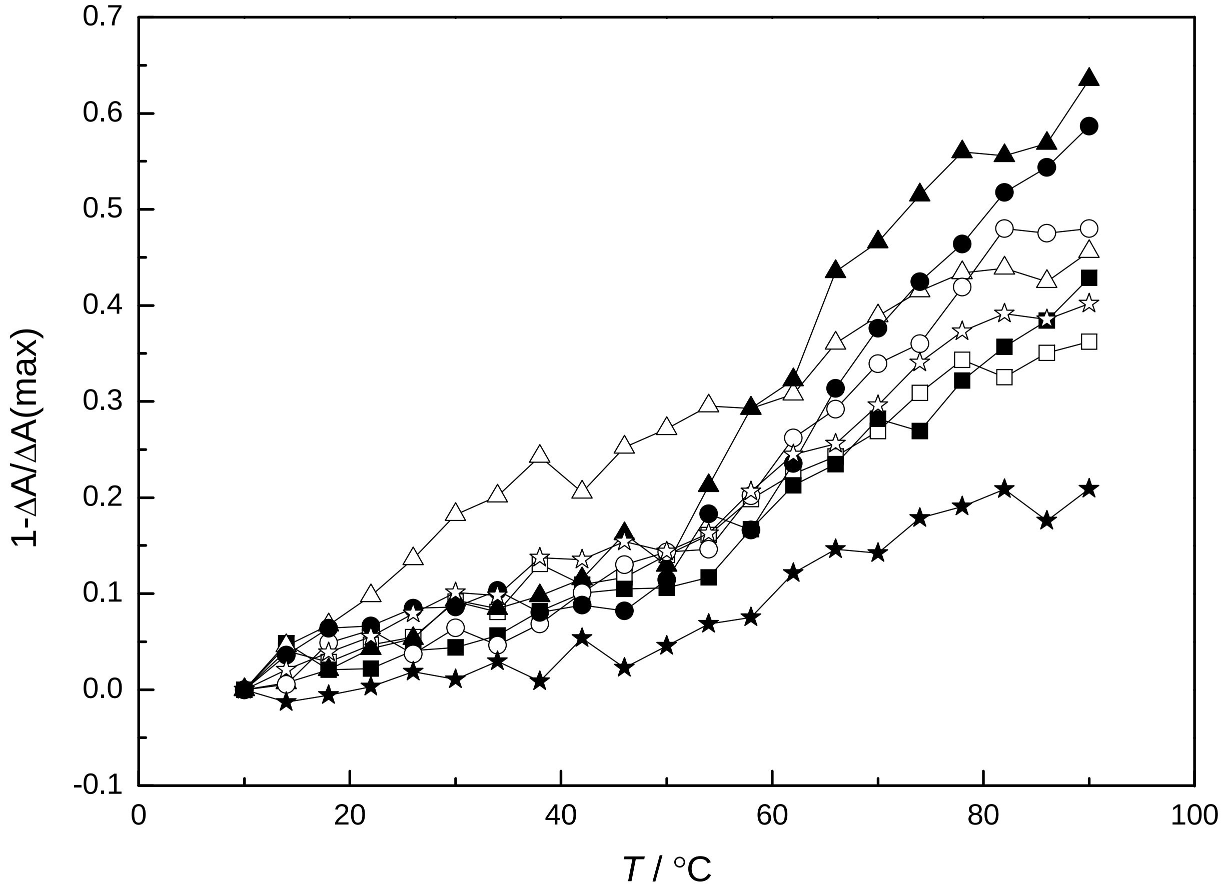

Figure S2. Temperature vs. 1- ΔA/ΔA(max) profiles of the heteroduplexes formed between **7X** and DNA oligonucleotides in the presence and absence of Pd^{2+}; scan range 220 – 320 nm; $I(NaClO_4)$ = 0.1 M; pH = 7.4. (□) **7X**:**10C**, (■) **7X**:**10C** + Pd^{2+}, (○) **7X**:**10G**, (●) **7X**:**10G** + Pd^{2+}, (☆) **7X**:**10T**, (★) **7X**:**10T** + Pd^{2+}, (△) **7X**:**10A**, (▲) **7X**:**10A** + Pd^{2+}.

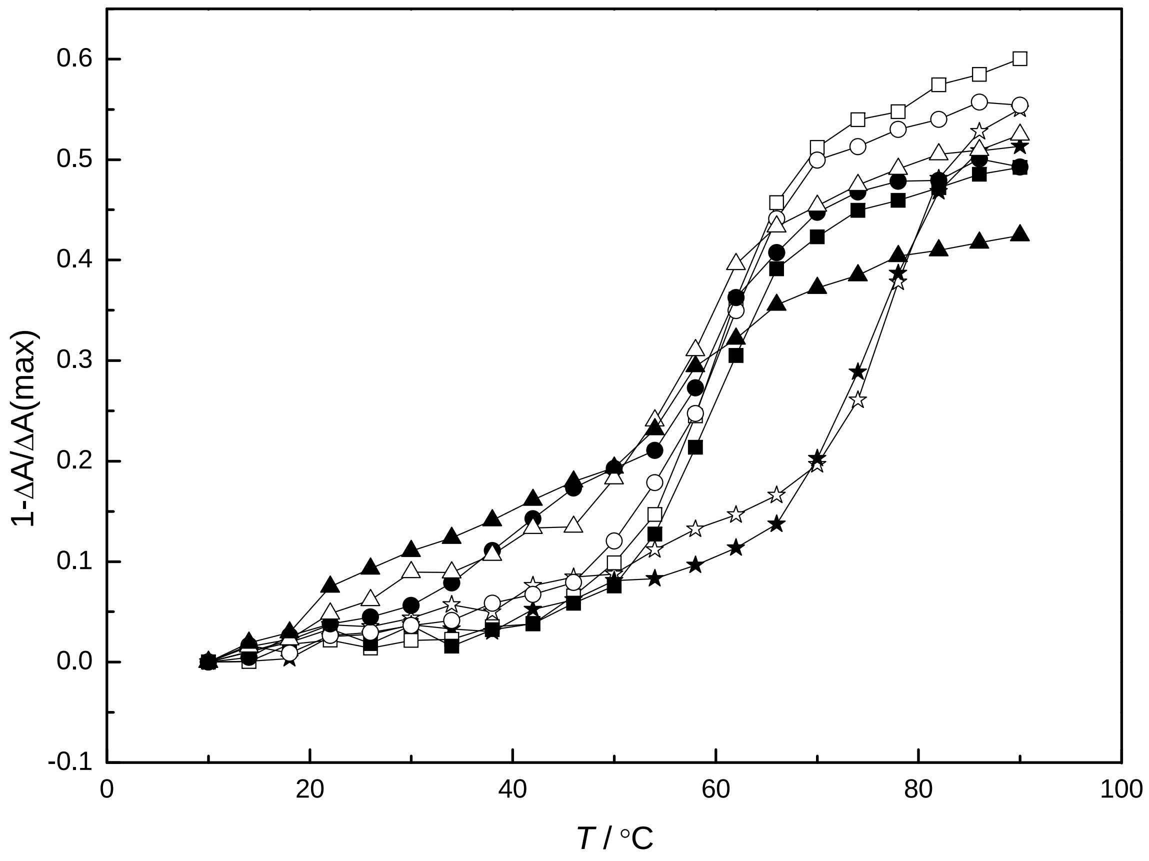

Figure S3. Temperature vs. 1- ΔA/ΔA(max) profiles of the homoduplexes formed between **7A** and 2′-*O*-methyl-RNA oligonucleotides in the presence and absence of Pd^{2+}; scan range 220 – 320 nm; $I(NaClO_4)$ = 0.1 M; pH = 7.4. (□) **7A**:**8C**, (■) **7A**:**8C** + Pd^{2+}, (○) **7A**:**8G**, (●) **7A**:**8G** + Pd^{2+}, (☆) **7A**:**8U**, (★) **7A**:**8U** + Pd^{2+}, (△) **7A**:**8A**, (▲) **7A**:**8A** + Pd^{2+}.

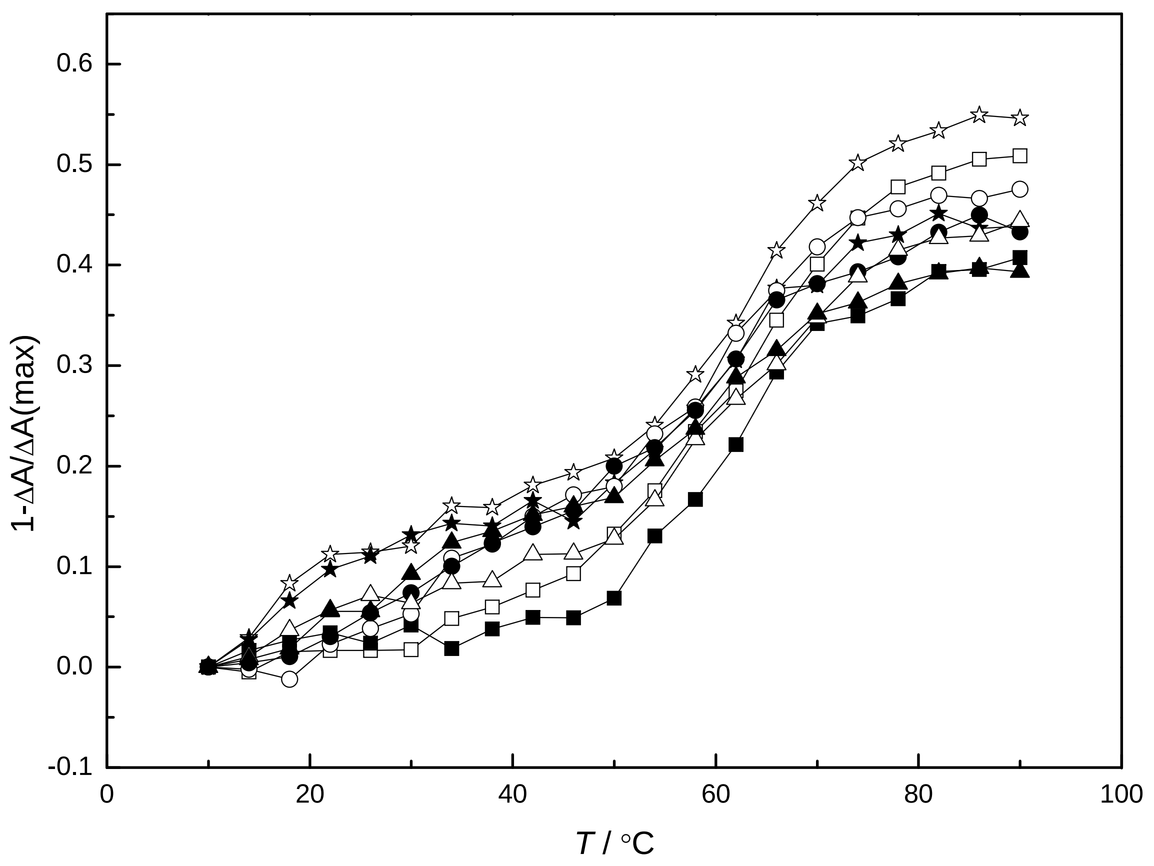

Figure S4. Temperature vs. 1- ΔA/ΔA(max) profiles of the homoduplexes formed between **7A** and 2′-*O*-methyl-RNA oligonucleotides in the presence and absence of Pd^{2+}; scan range 220 – 320 nm; *I*($NaClO_4$) = 0.1 M; pH = 7.4. (□) **7A**:**8C**, (■) **7A**:**8C** + Pd^{2+}, (○) **7A**:**8G**, (●) **7A**:**8G** + Pd^{2+}, (☆) **7A**:**8U**, (★) **7A**:**8U** + Pd^{2+}, (△) **7A**:**8A**, (▲) **7A**:**8A** + Pd^{2+}.

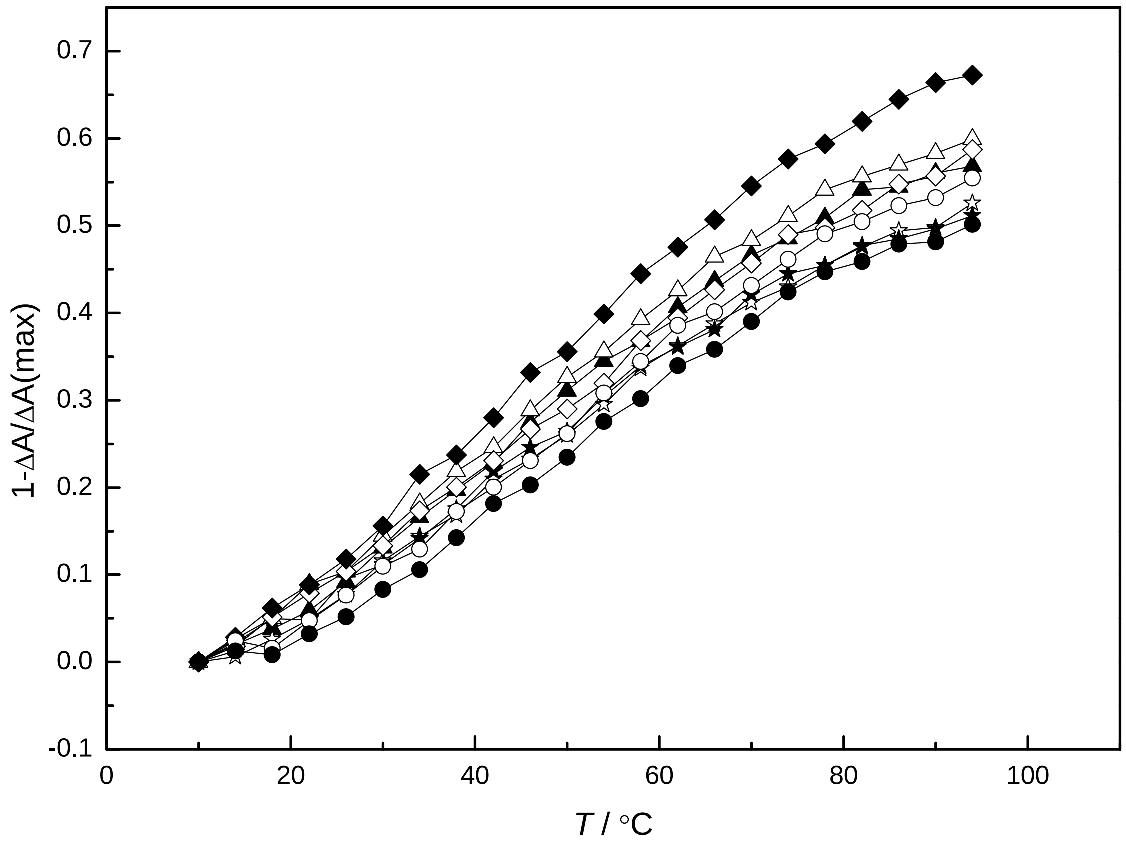

Figure S5. Temperature vs. 1- ΔA/ΔA(max) profiles of the mismatched homoduplexes formed between **7A** and 2´-*O*-methyl-RNA oligonucleotides in the presence and absence of Pd^{2+}; scan range 220 – 320 nm; *I*($NaClO_4$) = 0.1 M; pH = 7.4. (◇) **7A**:**9C**, (◆) **7A**:**9C** + Pd^{2+}, (△) **7A**:**9A**, (▲)**7A**:**9A** + Pd^{2+}, (○) **7A**:**9G**, (●) **7A**:**9G** + Pd^{2+}, (☆) **7A**:**9U**, (★) **7A**:**9U** + Pd^{2+}.

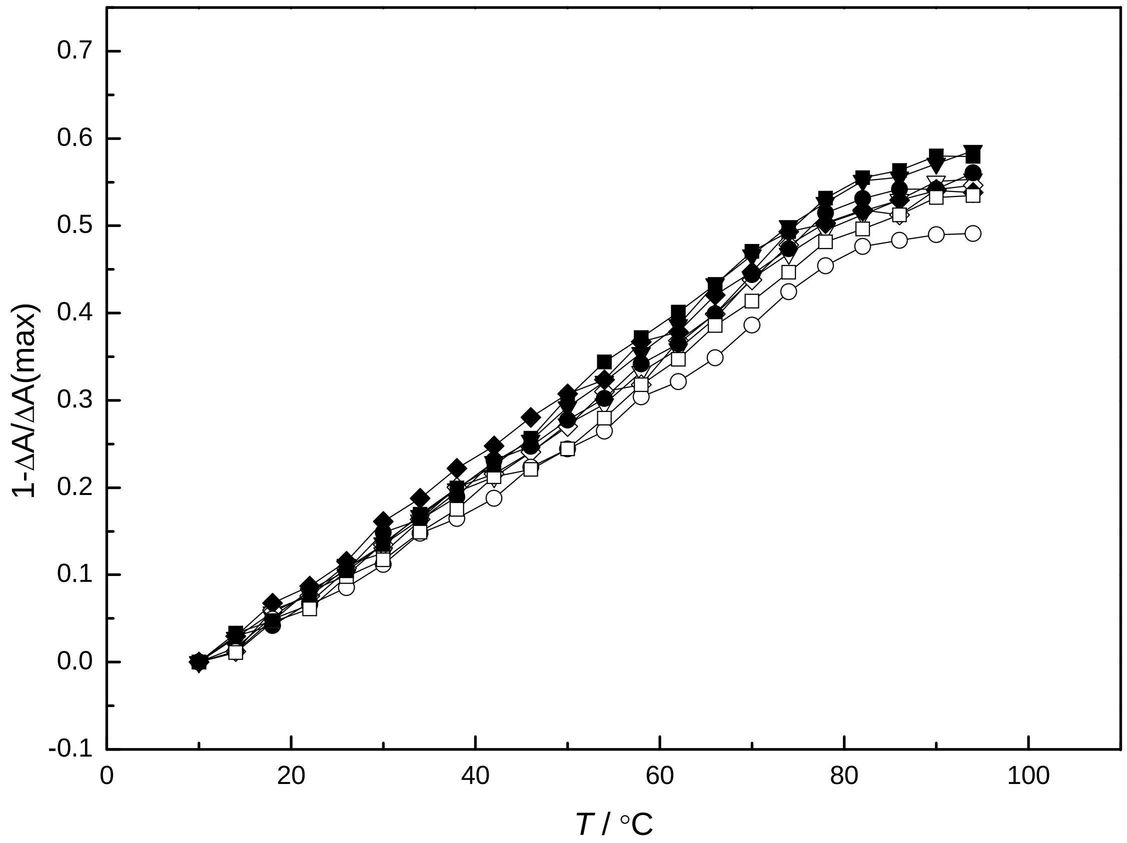

Figure S6. Temperature vs. 1- ΔA/ΔA(max) profiles of the mismatched homoduplexes formed between **7A** and 2′-*O*-methyl-RNA oligonucleotides in the presence and absence of Pd^{2+}; scan range 220 – 320 nm; $I(NaClO_4)$ = 0.1 M; pH = 7.4. (□) **7X**:**9C**, (■) **7X**:**9C** + Pd^{2+}, (▽) **7X**:**9A**, (▼) **7X**:**9A** + Pd^{2+}, (○) **7X**:**9G**, (●) **7X**:**9G** + Pd^{2+} , (◇) **7X**:**9U**, (◆) **7X**:**9U** + Pd^{2+}.

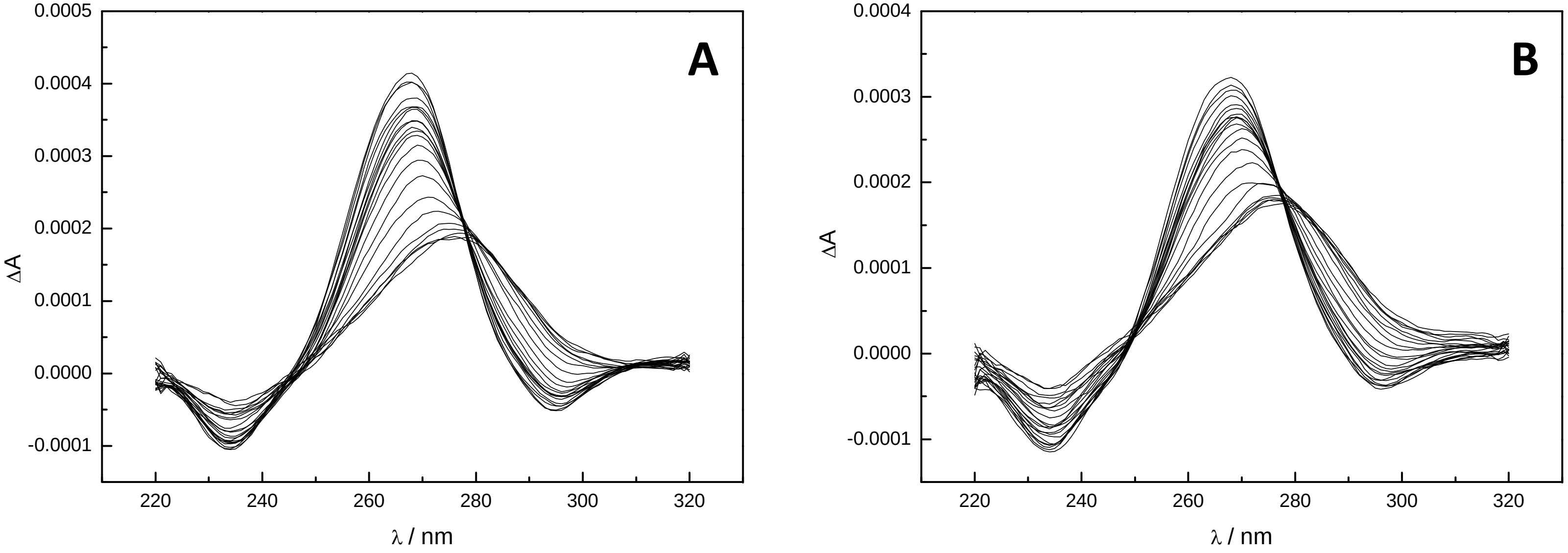

Figure S7. CD spectra of the homoduplex **7X**:**8U** in the absence (A) and presence (B) of Pd^{2+}; $I(NaClO_4) = 0.1$ mol L^{-1}, pH = 7.4.

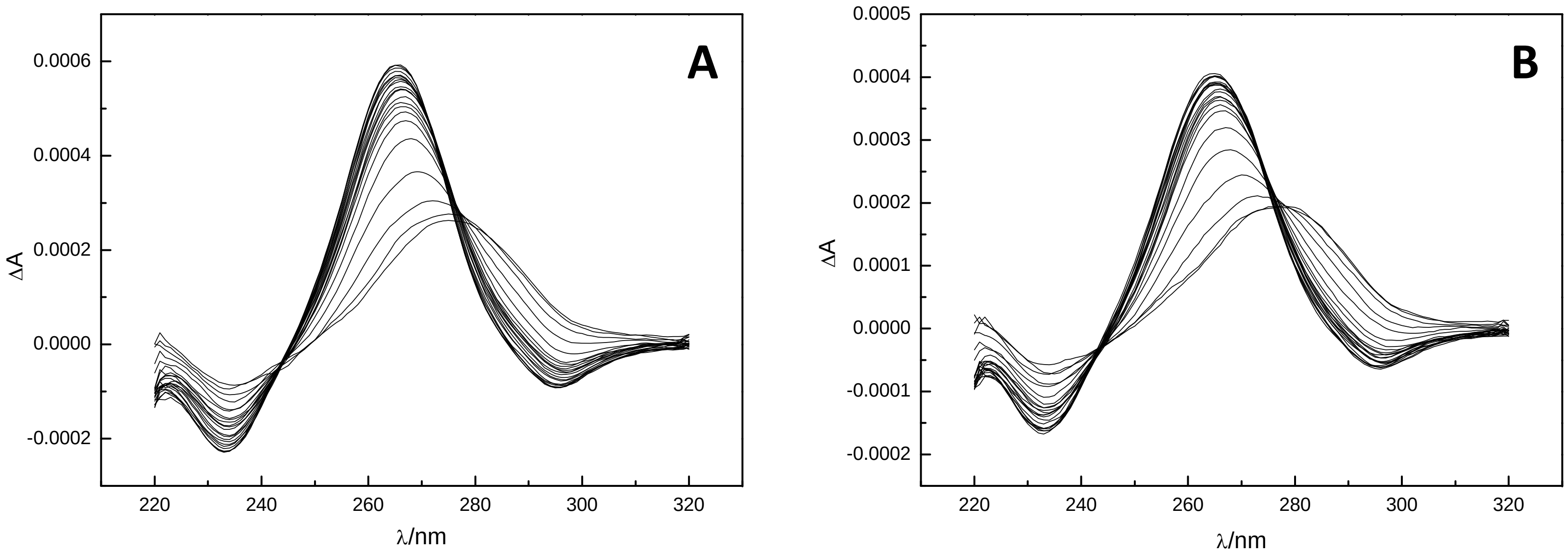

Figure S8. CD spectra of the homoduplex **7A**:**8U** in the absence (A) and presence (B) of Pd^{2+}; $I(NaClO_4)$ = 0.1 mol L^{-1}, pH = 7.4.

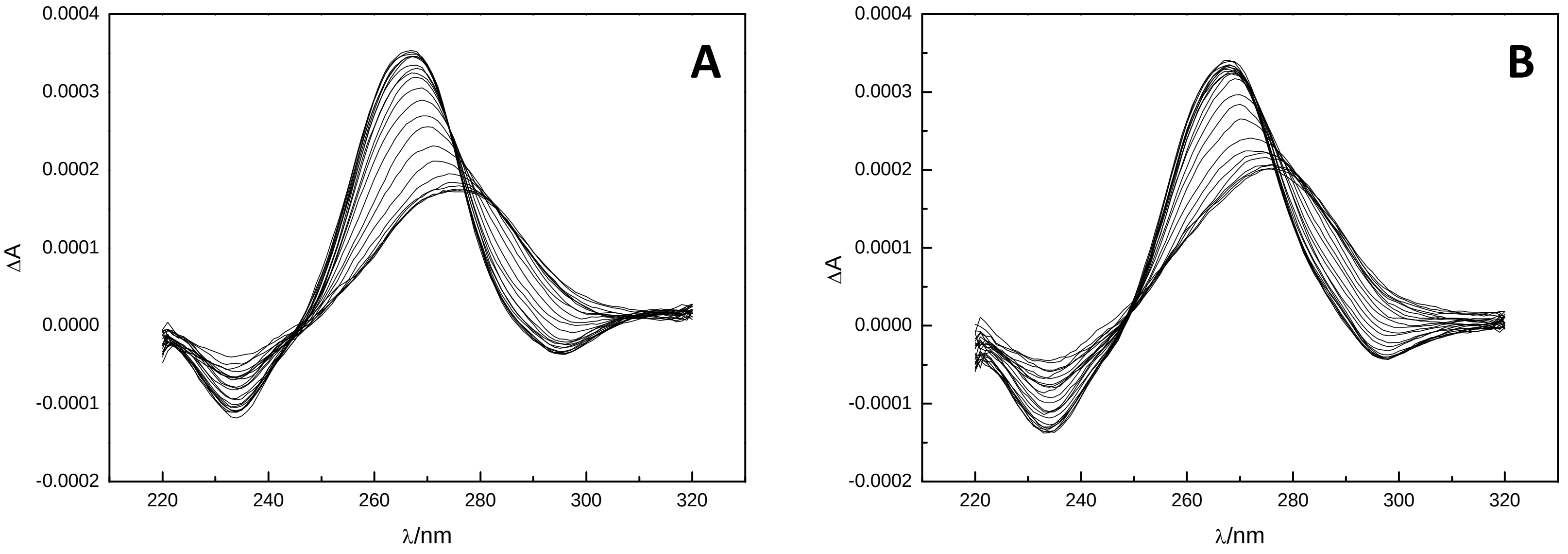

Figure S9. CD spectra of the homoduplex **7X**:**8C** in the absence (A) and presence (B) of Pd^{2+}; $I(NaClO_4) = 0.1$ mol L^{-1}, pH = 7.4.

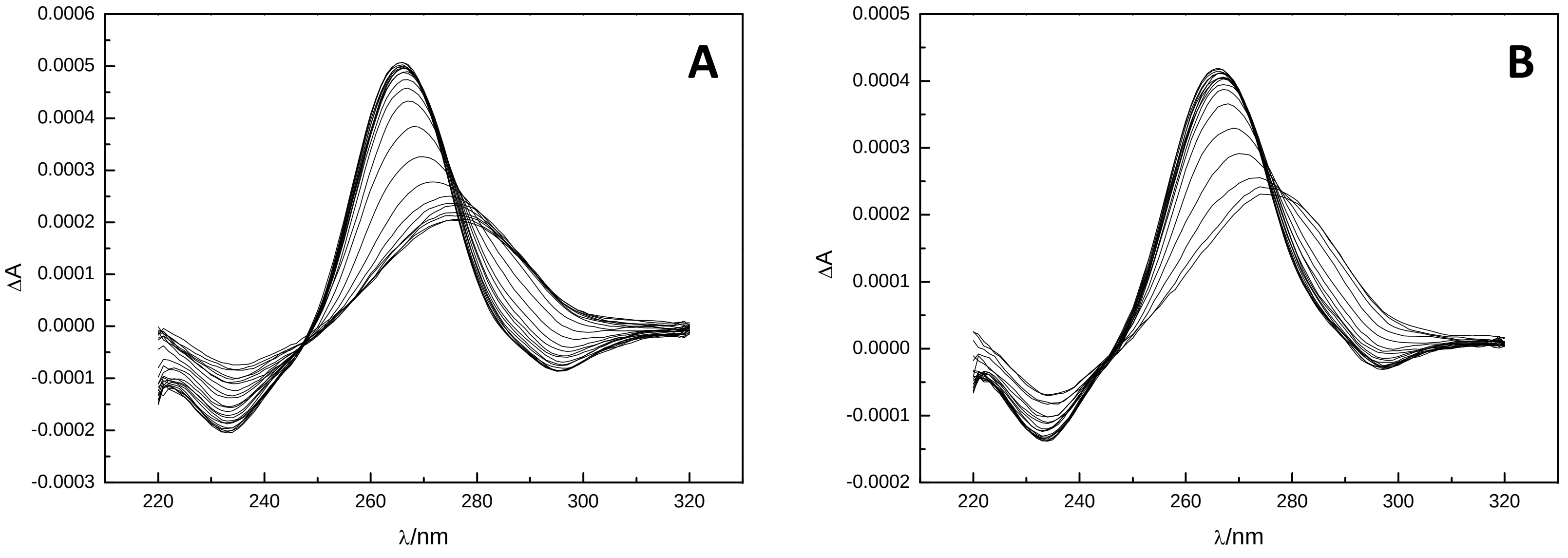

Figure S10. CD spectra of the homoduplex **7A**:**8C** in the absence (A) and presence (B) of Pd^{2+}; $I(NaClO_4)$ = 0.1 mol L^{-1}, pH = 7.4.

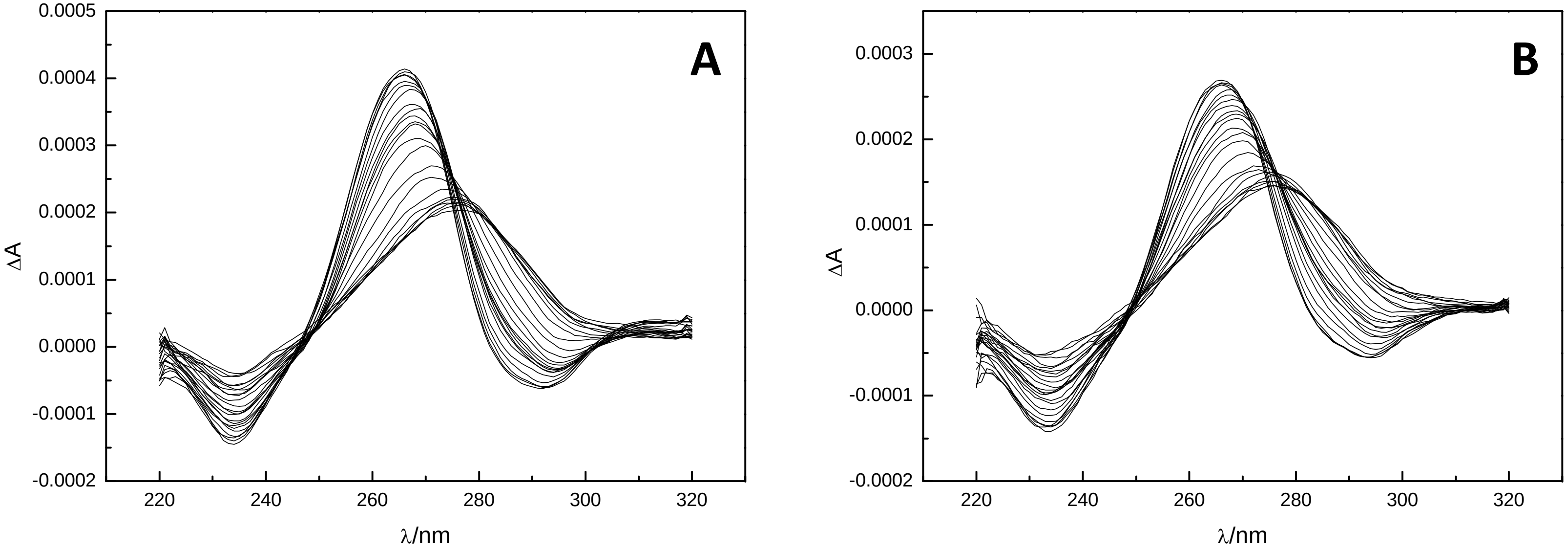

Figure S11. CD spectra of the homoduplex **7X**:**8G** in the absence (A) and presence (B) of Pd^{2+}; $I(NaClO_4)$ = 0.1 mol L^{-1}, pH = 7.4.

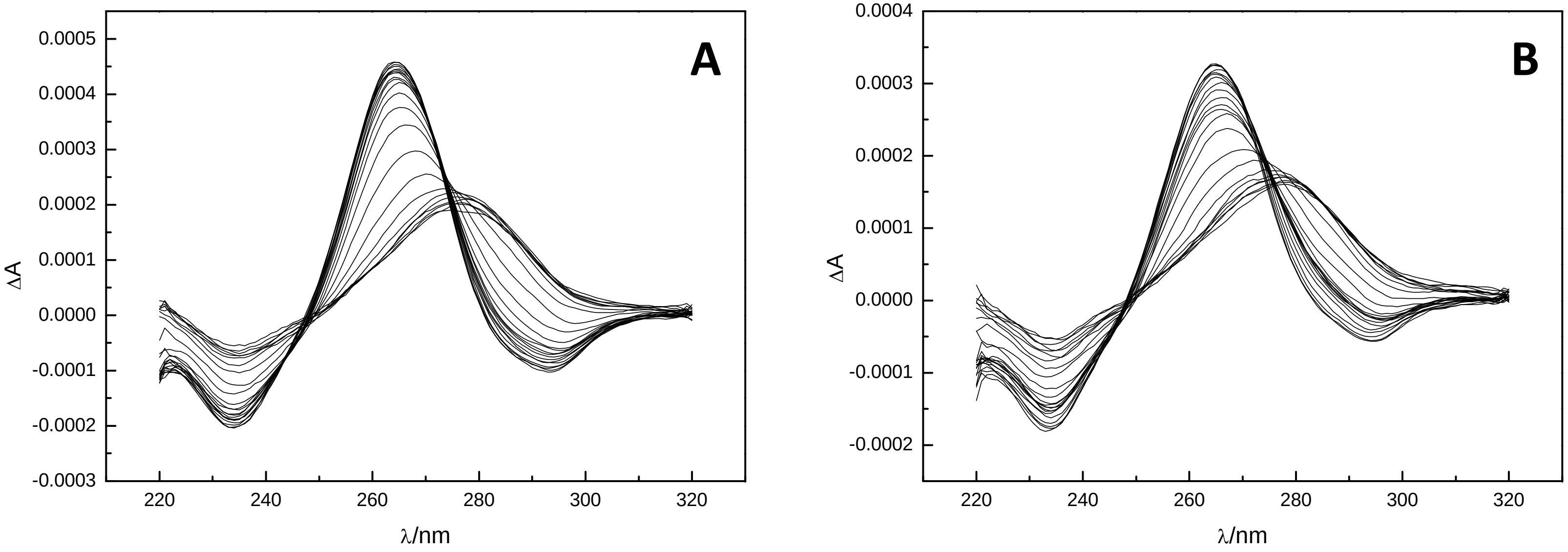

Figure S12. CD spectra of the homoduplex **7A**:**8G** in the absence (A) and presence (B) of Pd^{2+}; $I(NaClO_4)$ = 0.1 mol L^{-1}, pH = 7.4.

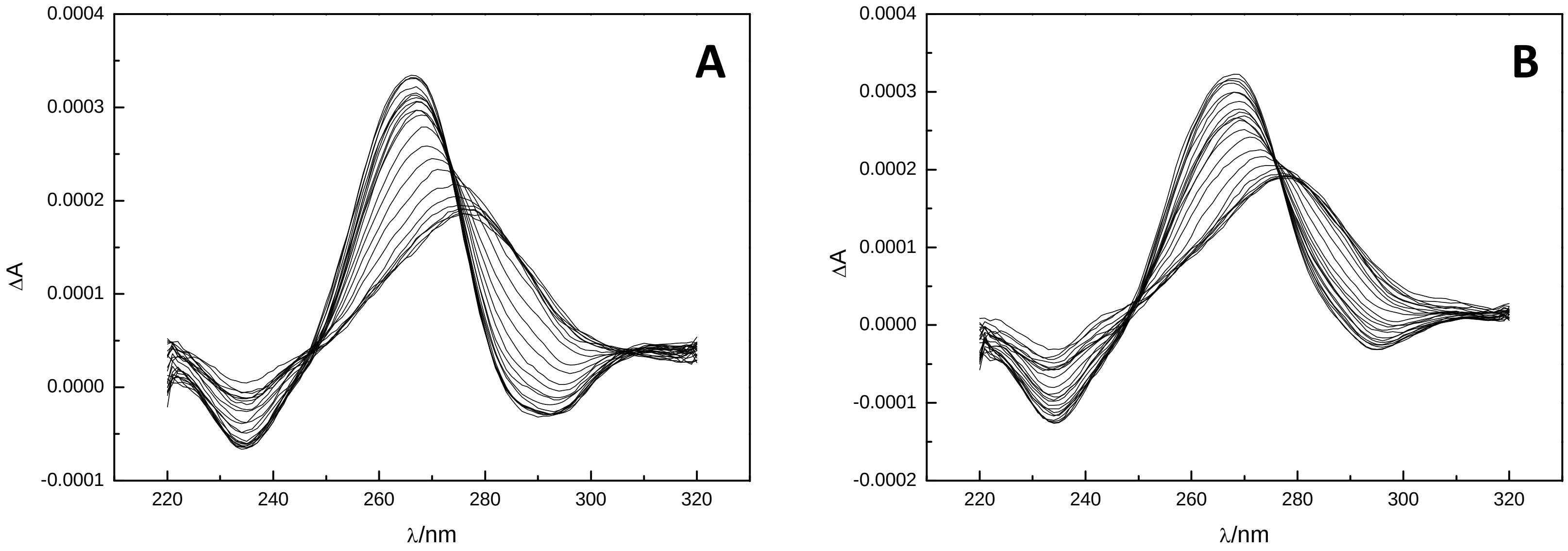

Figure S13. CD spectra of the homoduplex **7X**:**8A** in the absence (A) and presence (B) of Pd^{2+}; $I(NaClO_4) = 0.1$ mol L^{-1}, pH = 7.4.

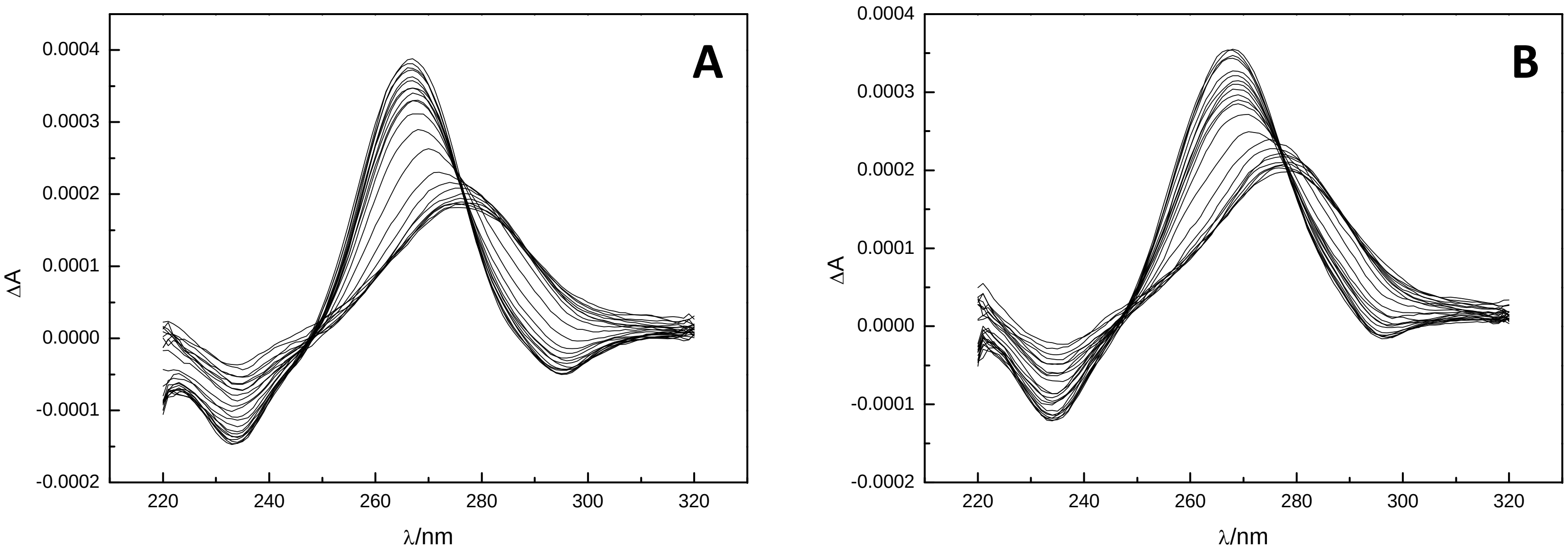

Figure S14. CD spectra of the homoduplex **7A**:**8A** in the absence (A) and presence (B) of Pd^{2+}; $I(NaClO_4) = 0.1$ mol L^{-1}, pH = 7.4.

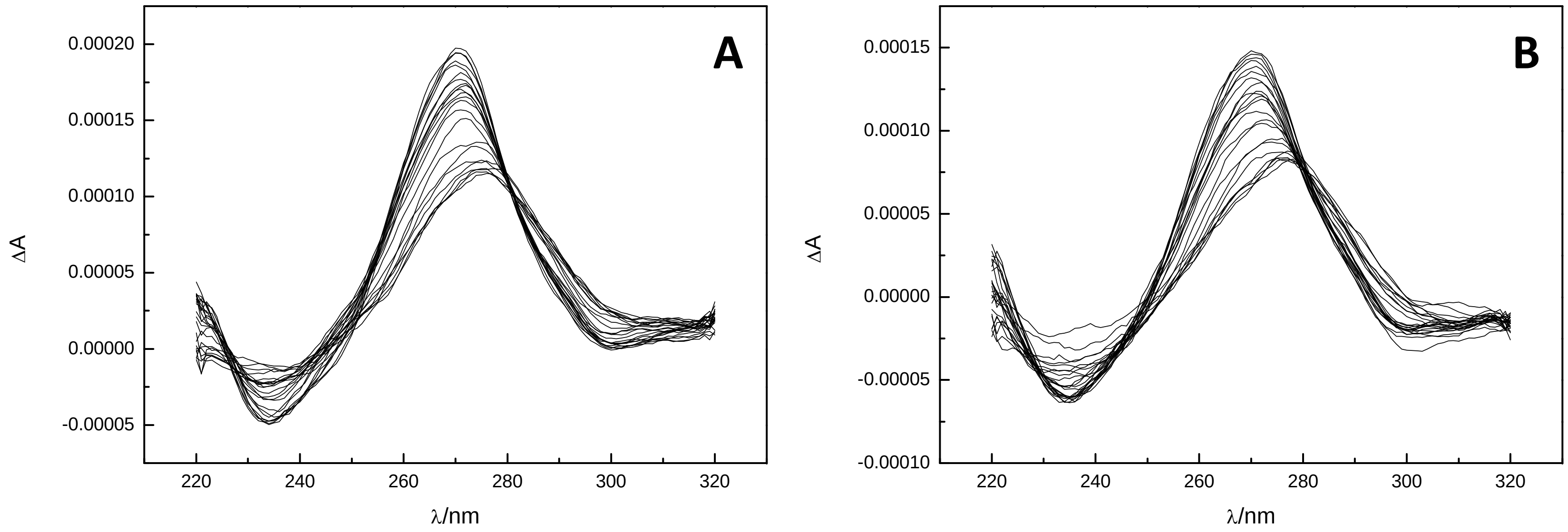

Figure S15. CD spectra of the heteroduplex **7X**:**10A** in the absence (A) and presence (B) of Pd^{2+}; $I(NaClO_4)$ = 0.1 mol L^{-1}, pH = 7.4.

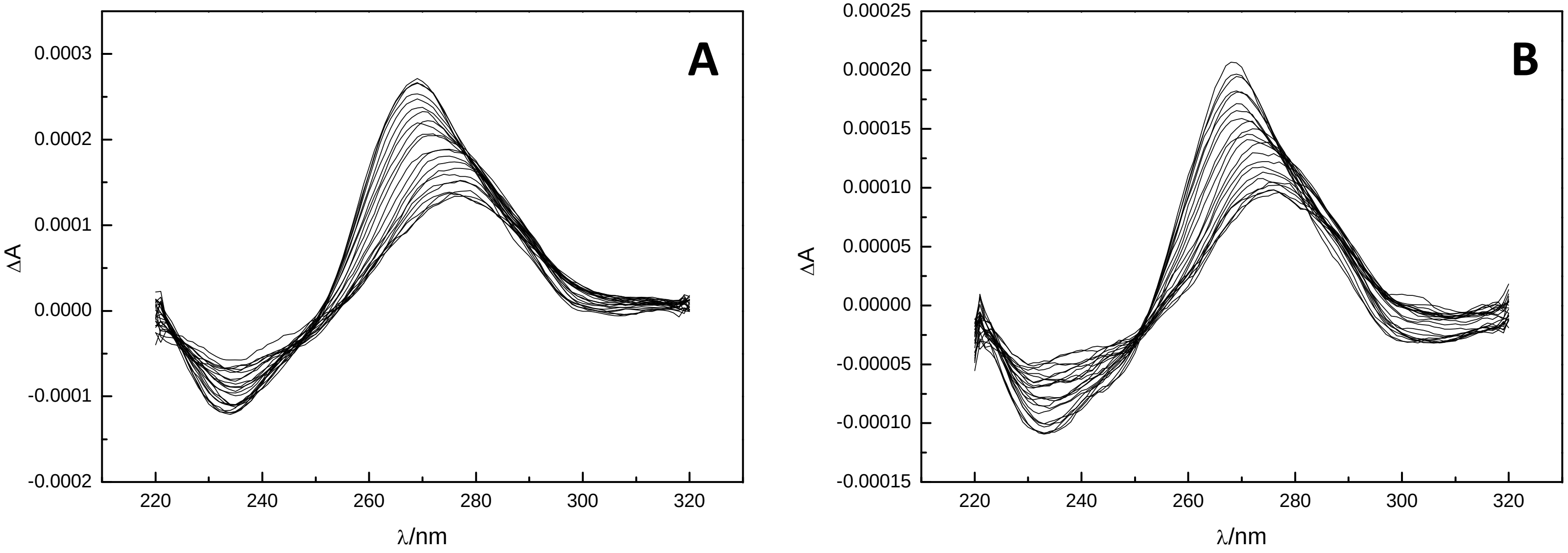

Figure S16. CD spectra of the heteroduplex **7A**:**10A** in the absence (A) and presence (B) of Pd^{2+}; $I(NaClO_4)$ = 0.1 mol L^{-1}, pH = 7.4.

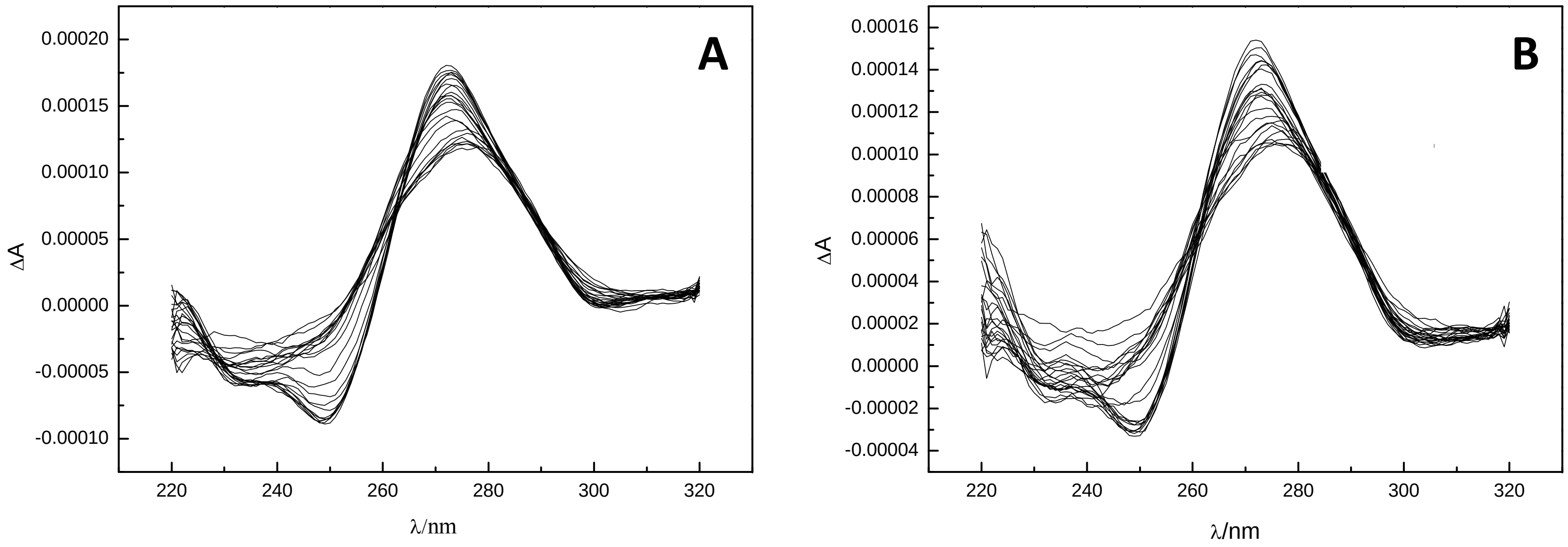

Figure S17. CD spectra of the heteroduplex **7X**:**10C** in the absence (A) and presence (B) of Pd^{2+}; $I(NaClO_4)$ = 0.1 mol L^{-1}, pH = 7.4.

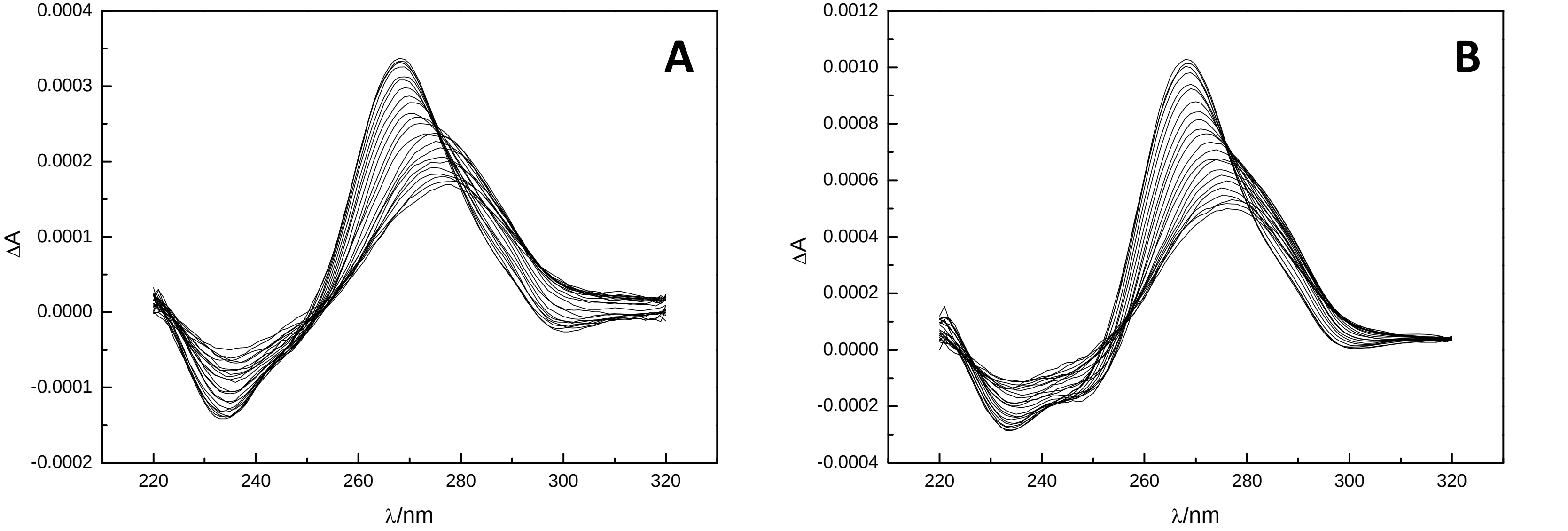

Figure S18. CD spectra of the heteroduplex **7A**:**10C** in the absence (A) and presence (B) of Pd^{2+}; $I(NaClO_4) = 0.1$ mol L^{-1}, pH = 7.4.

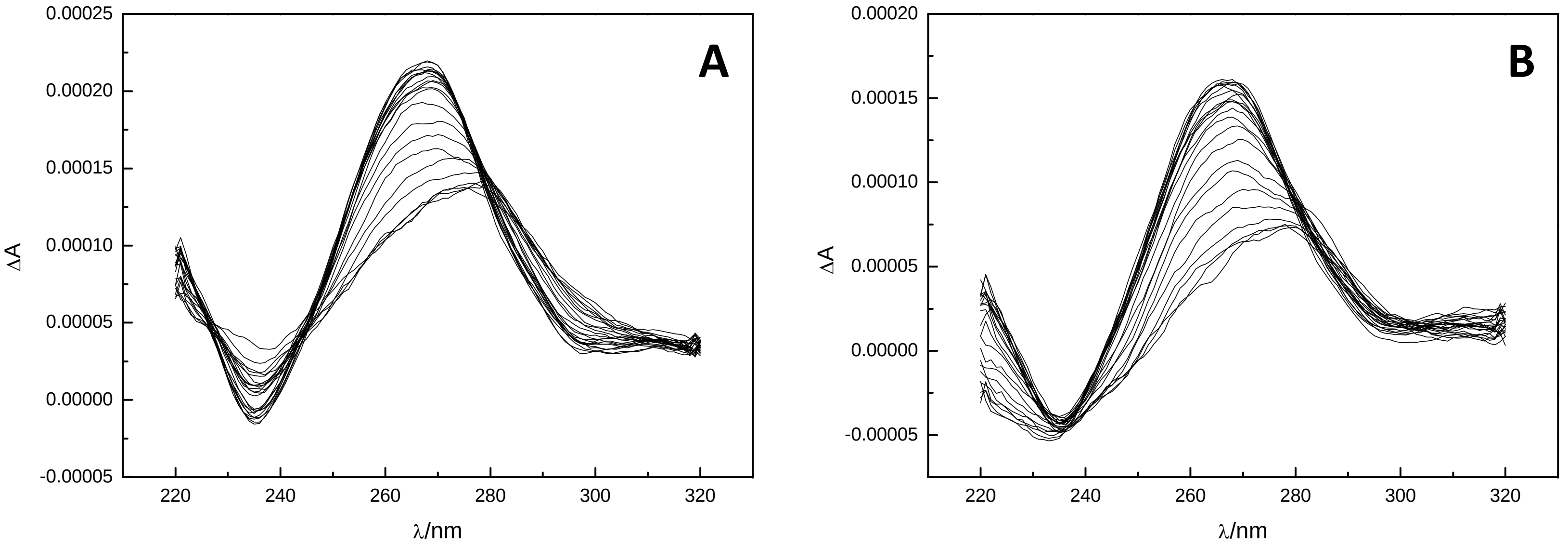

Figure S19. CD spectra of the heteroduplex **7X**:**10G** in the absence (A) and presence (B) of Pd^{2+}; $I(NaClO_4)$ = 0.1 mol L^{-1}, pH = 7.4.

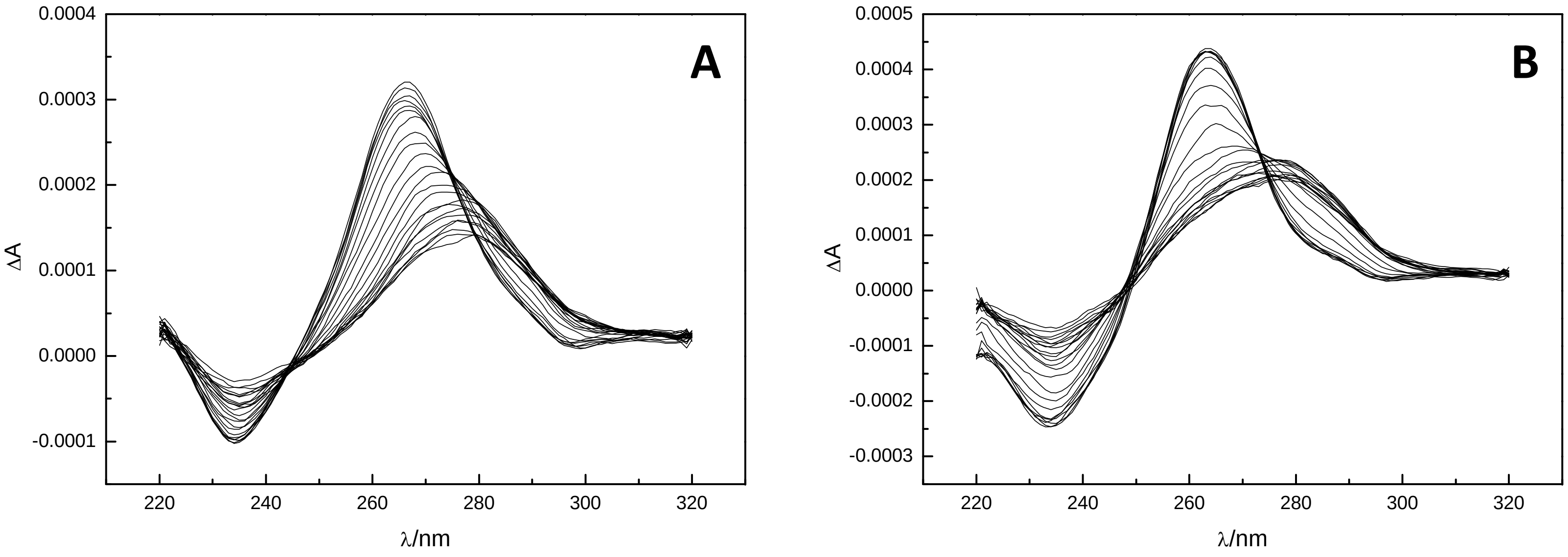

Figure S20. CD spectra of the heteroduplex **7A**:**10G** in the absence (A) and presence (B) of Pd^{2+}; $I(NaClO_4)$ = 0.1 mol L^{-1}, pH = 7.4.

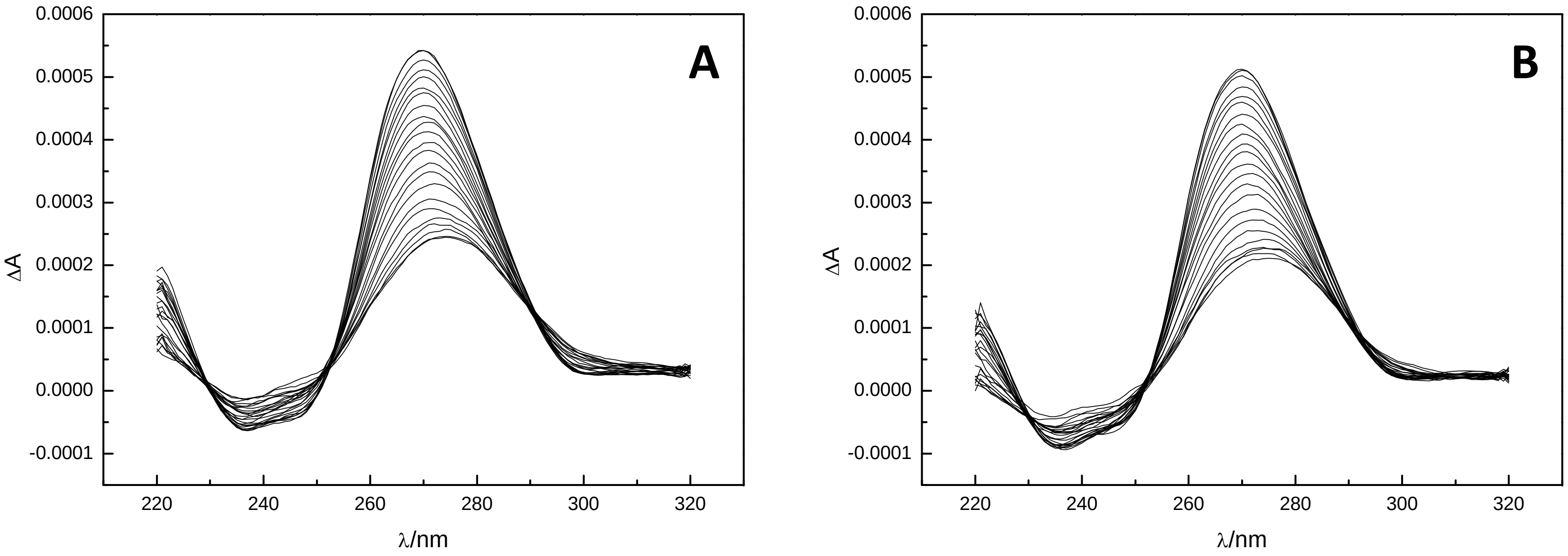

Figure S21. CD spectra of the mismatched homoduplex **7X**:**9A** in the absence (A) and presence (B) of Pd^{2+}; $I(NaClO_4)$ = 0.1 mol L^{-1}, pH = 7.4.

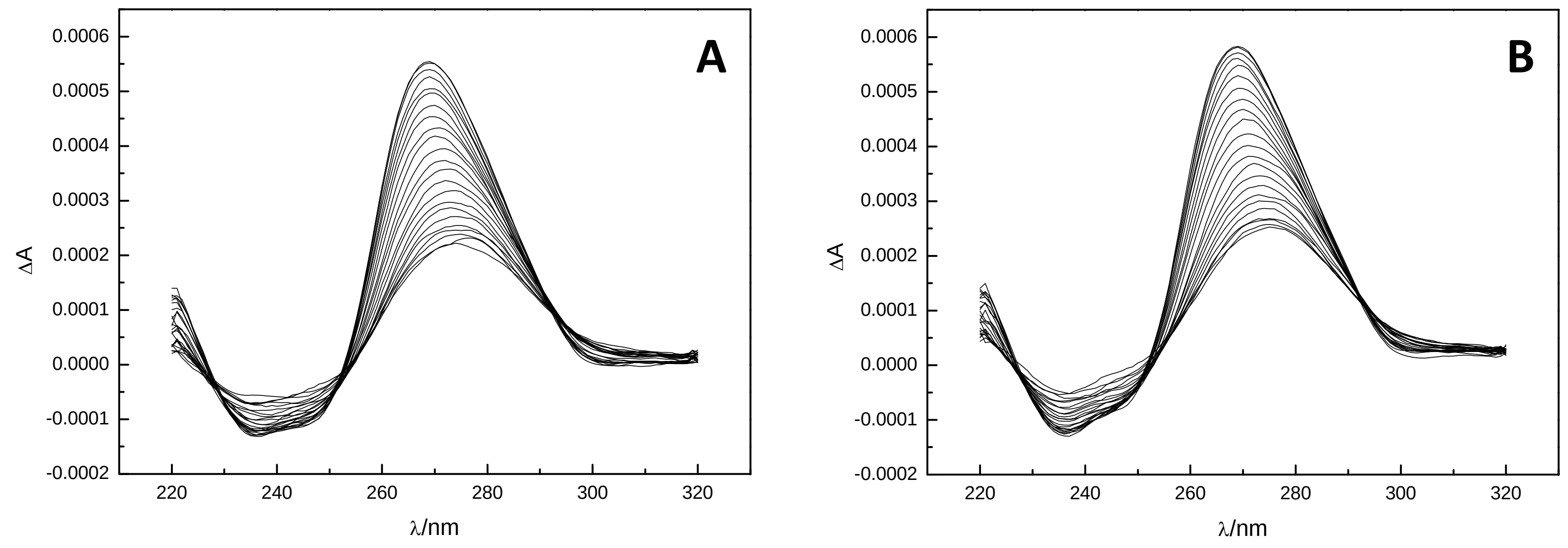

Figure S22. CD spectra of the mismatched homoduplex **7A**:**9A** in the absence (A) and presence (B) of Pd^{2+}; $I(NaClO_4) = 0.1$ mol L^{-1}, pH = 7.4.

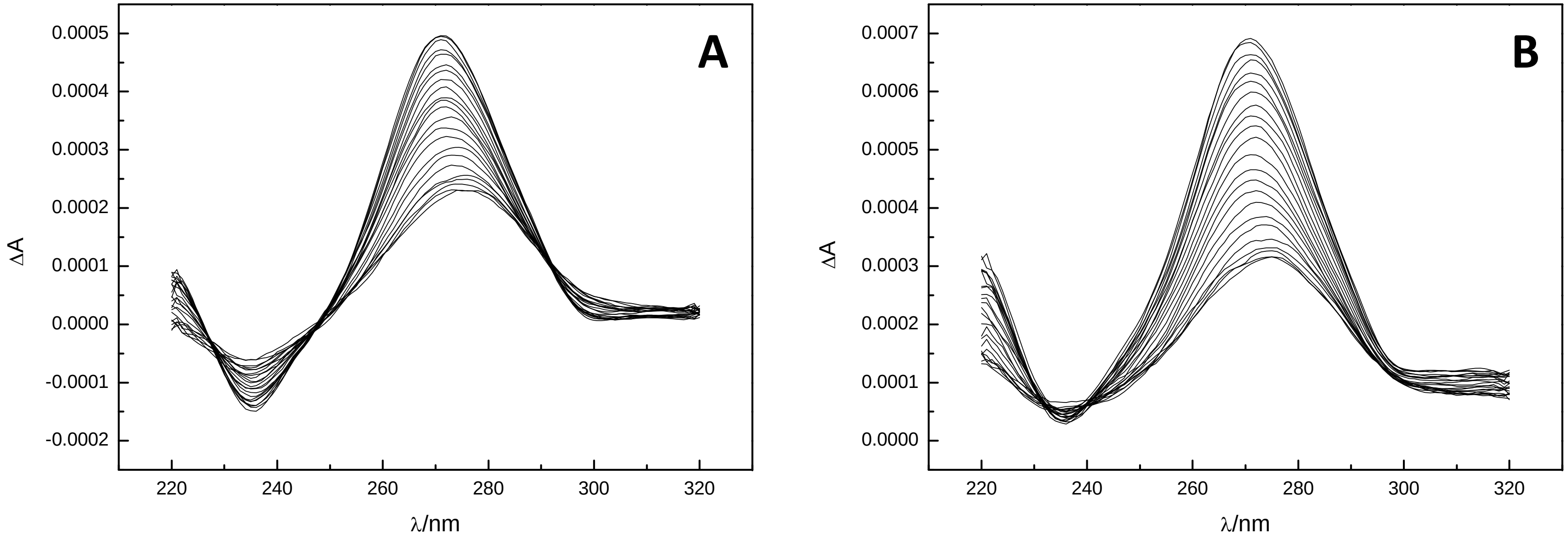

Figure S23. CD spectra of the mismatched homoduplex **7X**:**9C** in the absence (A) and presence (B) of Pd^{2+}; $I(NaClO_4)$ = 0.1 mol L^{-1}, pH = 7.4.

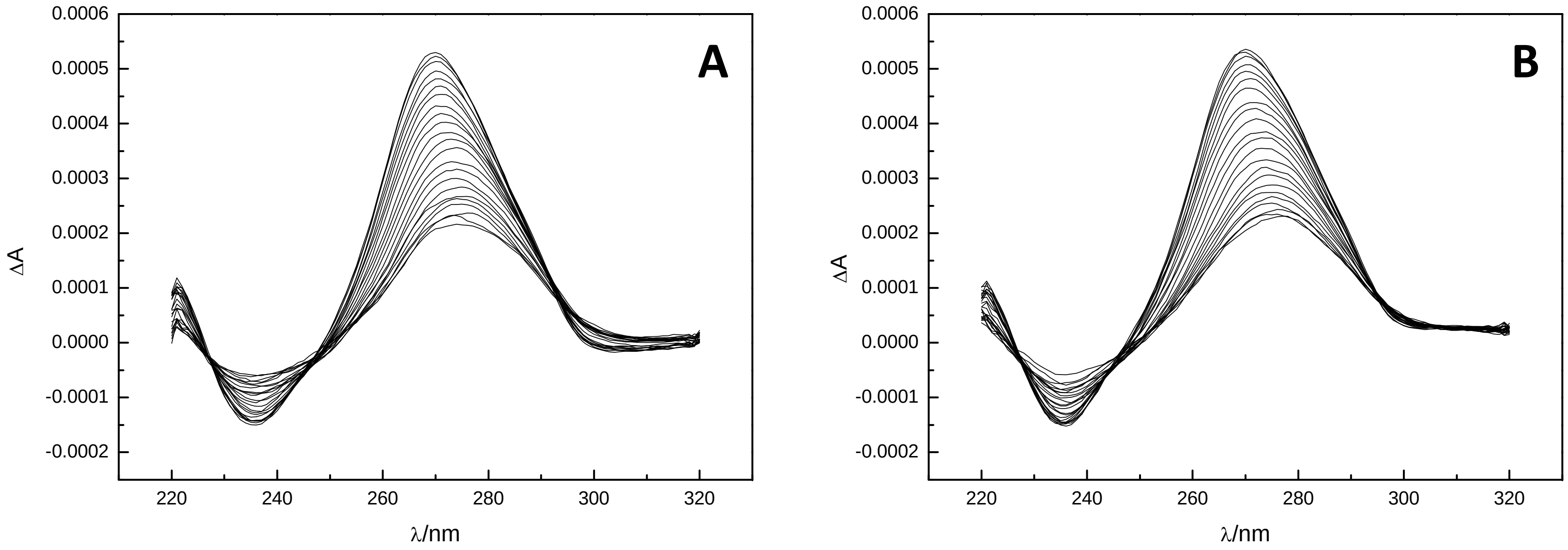

Figure S24. CD spectra of the mismatched homoduplex **7A**:**9C** in the absence (A) and presence (B) of Pd^{2+}; $I(NaClO_4) = 0.1$ mol L^{-1}, pH = 7.4.

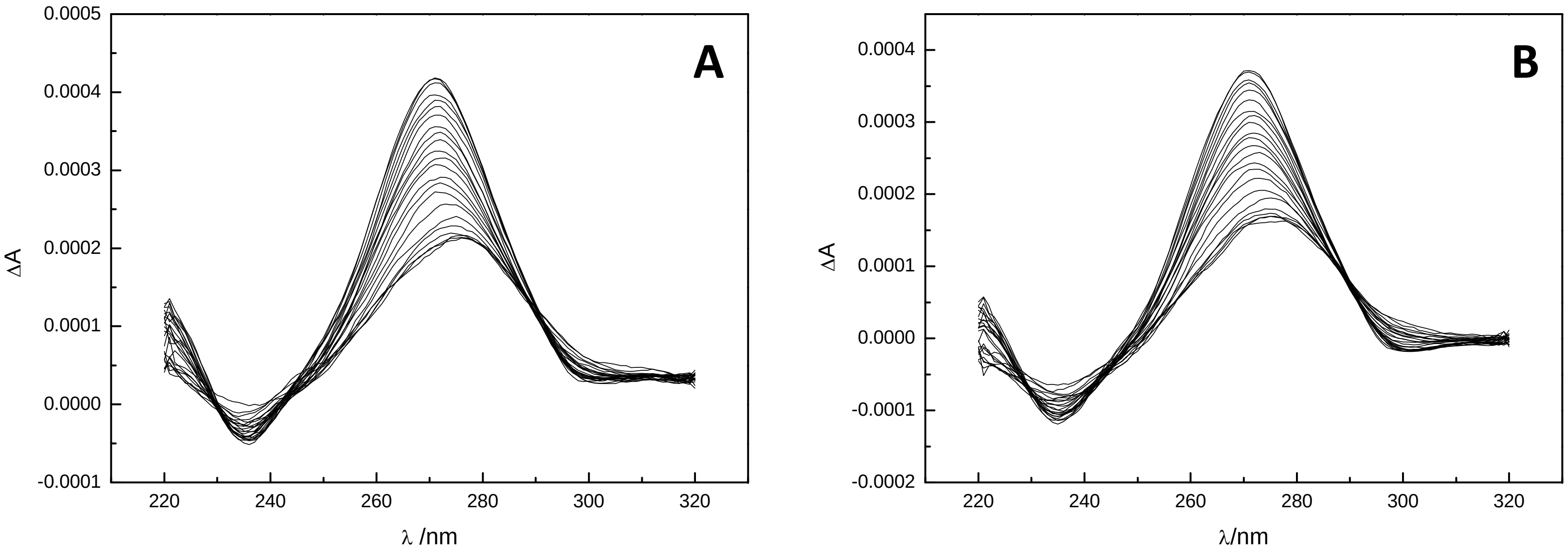

Figure S25. CD spectra of the mismatched homoduplex **7X**:**9G** in the absence (A) and presence (B) of Pd^{2+}; $I(NaClO_4)$ = 0.1 mol L^{-1}, pH = 7.4.

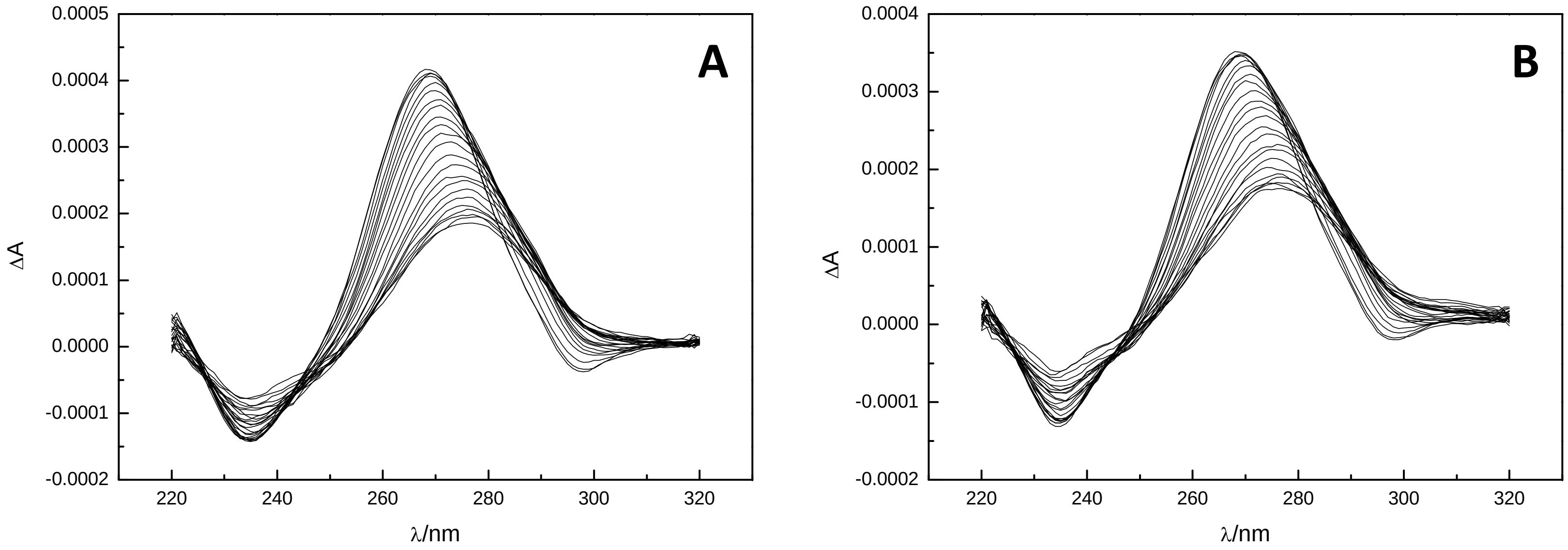

Figure S26. CD spectra of the mismatched homoduplex **7A**:**9G** in the absence (A) and presence (B) of Pd^{2+}; $I(NaClO_4)$ = 0.1 mol L^{-1}, pH = 7.4.

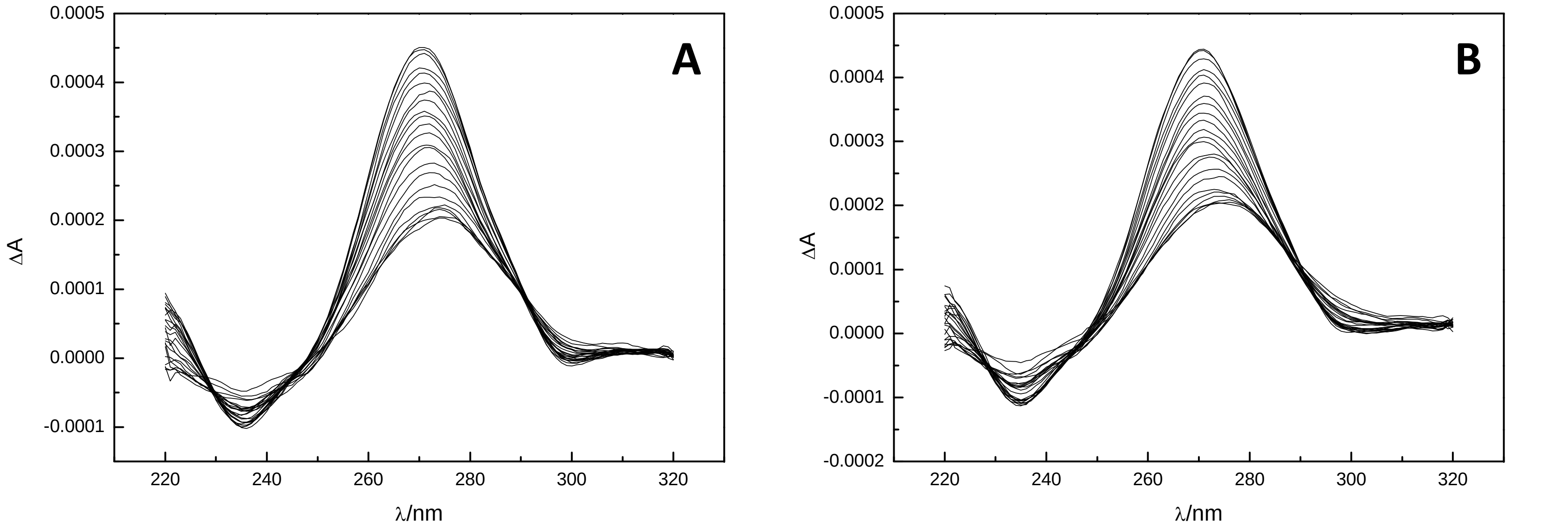

Figure S27. CD spectra of the mismatched homoduplex **7X**:**9U** in the absence (A) and presence (B) of Pd^{2+}; $I(NaClO_4)$ = 0.1 mol L^{-1}, pH = 7.4.

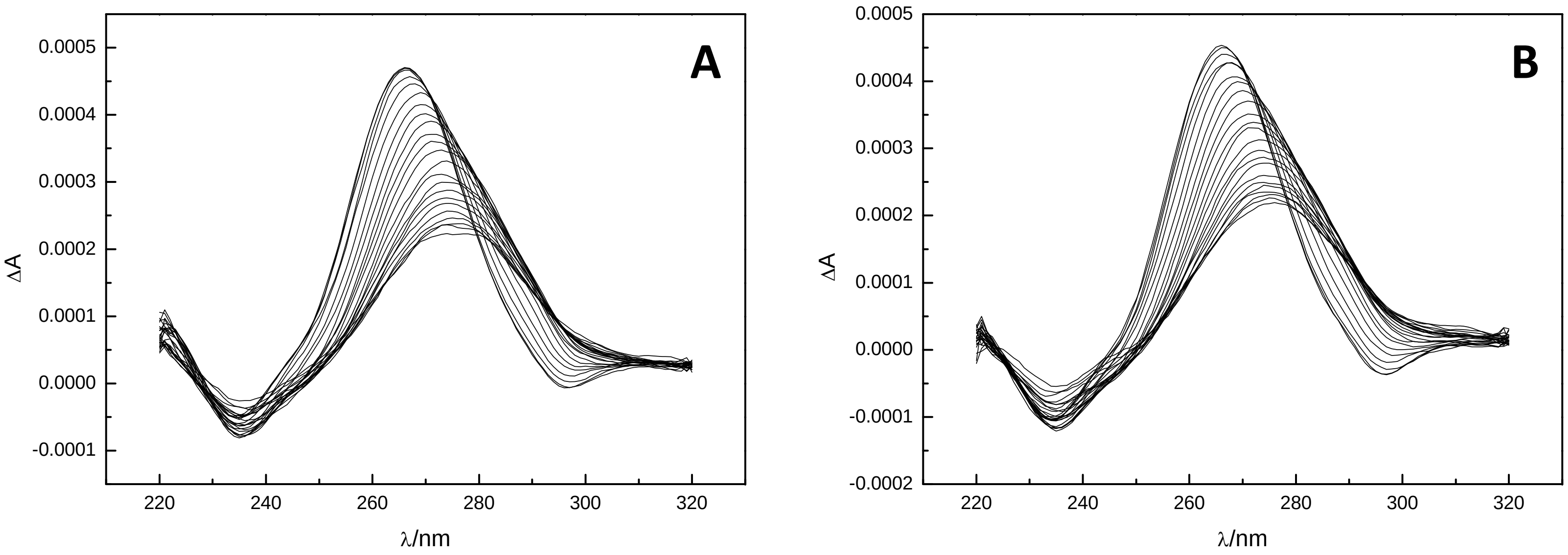

Figure S28. CD spectra of the mismatched homoduplex **7A**:**9U** in the absence (A) and presence (B) of Pd^{2+}; $I(NaClO_4)$ = 0.1 mol L^{-1}, pH = 7.4.

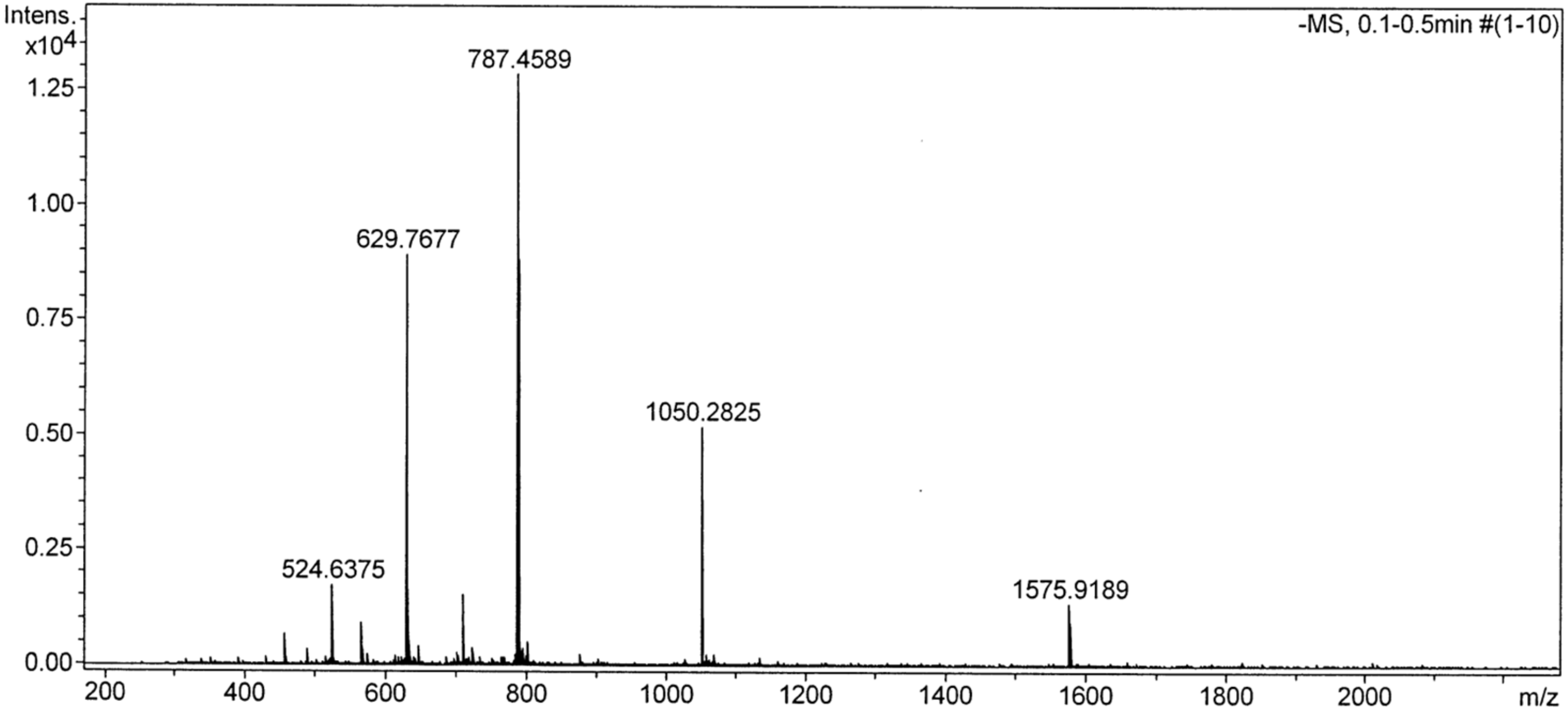

Figure S29. ESI$^-$ mass spectrum of the modified oligonucleotide **7X**.
